# Efficient dilution-to-extinction isolation of novel virus–host model systems for fastidious heterotrophic bacteria

**DOI:** 10.1038/s41396-020-00872-z

**Published:** 2021-01-25

**Authors:** Holger H. Buchholz, Michelle L. Michelsen, Luis M. Bolaños, Emily Browne, Michael J. Allen, Ben Temperton

**Affiliations:** 1 School of Biosciences, University of Exeter, Exeter, UK; 2 Plymouth Marine Laboratory, Plymouth, UK

**Keywords:** Bacteriophages, Microbial ecology

## Abstract

Microbes and their associated viruses are key drivers of biogeochemical processes in marine and soil biomes. While viruses of phototrophic cyanobacteria are well-represented in model systems, challenges of isolating marine microbial heterotrophs and their viruses have hampered experimental approaches to quantify the importance of viruses in nutrient recycling. A resurgence in cultivation efforts has improved the availability of fastidious bacteria for hypothesis testing, but this has not been matched by similar efforts to cultivate their associated bacteriophages. Here, we describe a high-throughput method for isolating important virus–host systems for fastidious heterotrophic bacteria that couples advances in culturing of hosts with sequential enrichment and isolation of associated phages. Applied to six monthly samples from the Western English Channel, we first isolated one new member of the globally dominant bacterial SAR11 clade and three new members of the methylotrophic bacterial clade OM43. We used these as bait to isolate 117 new phages, including the first known siphophage-infecting SAR11, and the first isolated phage for OM43. Genomic analyses of 13 novel viruses revealed representatives of three new viral genera, and infection assays showed that the viruses infecting SAR11 have ecotype-specific host ranges. Similar to the abundant human-associated phage ɸCrAss001, infection dynamics within the majority of isolates suggested either prevalent lysogeny or chronic infection, despite a lack of associated genes, or host phenotypic bistability with lysis putatively maintained within a susceptible subpopulation. Broader representation of important virus–host systems in culture collections and genomic databases will improve both our understanding of virus–host interactions, and accuracy of computational approaches to evaluate ecological patterns from metagenomic data.

## Introduction

It is estimated that viral predation kills ~15% of bacterial cells in marine surface water each day [1] and is a major contributor to nutrient recycling via the viral shunt, where marine viruses make cell-bound nutrients available to the neighbouring microbial community through viral lysis of host cells [2, 3]. Viruses are key players in the modulation of carbon fluxes across the oceans (150 Gt/yr), increasing particle aggregation and sinking to depth [2, 4], and accounting for 89% of the variance in carbon export from surface water to the deep ocean [5]. Viruses alter host metabolism through auxiliary metabolic genes (AMGs), increasing and altering the cellular carbon intake of infected cells [6]. Virus–host interactions also increase co-evolutionary rates of both predator and prey via Red Queen dynamics [7, 8]. While recent metagenomic advances have provided major insight into global viral diversity and abundance [9–12], mechanistic understanding of virus–host interactions in ecologically important taxa is reliant on experimental co-culturing of model systems. In cyanobacteria, such systems have shown that viruses increase the duration of photosynthetic function [13] and can inhibit CO_2_ fixation, providing direct evidence that viruses of abundant phototrophs play an important role in nutrient cycling and global carbon budgets [14]. Furthermore, isolation of new viruses provides complete or near-complete viral genomes, with concrete evidence of known hosts. Such systems are critical to the development, ground-truthing and application of computational methods to identify and classify viral genomes in metagenomic data (e.g. VirSorter [15], VirFinder [16] and MARVEL [17]), quantify boundaries for viral populations [12, 18] and genera (VConTACT2 [19]), understand the importance of AMGs in altering nutrient flux in natural communities [20] and to predict host ranges of uncultured viruses in metagenomic data (e.g. WIsH [21, 22]).

Viruses of primary producers, such as cyanophages, are both well-represented with model systems and well-studied in the laboratory. In contrast, virus–host model systems for similarly important and abundant marine heterotrophic bacteria are rare. Isolated viruses infecting heterotrophs are heavily biased towards those with fast-growing, copiotrophic hosts that grow readily on solid agar, enabling the use of plaque assays for viral isolation. Such systems are not representative of the vast majority of heterotrophs in nutrient-limited soil and aquatic environments, which are dominated by slow-growing, oligotrophic taxa with few regulatory mechanisms and complex auxotrophies that limit growth on solid media [23–26]. Advances in dilution-to-extinction culturing of ecologically important hosts have enabled the cultivation of many fastidious bacterial taxa that are not amenable to growth on solid media from soil [27], marine [28, 29] and freshwater environments [30]. Without plaque assays to facilitate isolation and purification of viral isolates, cultivation of viruses infecting fastidious taxa in liquid media is challenging, and further exacerbated by the slow growth rates and complex nutrient requirements of their hosts. The paucity of such model systems introduces significant bias in our understanding of viral influence on global carbon biogeochemical cycles. Therefore, it is important that the efforts to isolate heterotrophic bacterial taxa for experimentation and synthetic ecology are matched by efforts to isolate their associated viruses.

Here, we adapted recent advances in dilution-to-extinction culturing of hosts [29], and protocols to isolate viruses from liquid media [31] to improve the efficiency of cultivating novel virus–host systems for fastidious taxa: First, we selected the ecologically significant SAR11 and OM43 heterotrophic marine clades as models for viral isolation; second, we used sequential enrichment of viruses from natural communities on target hosts to improve the rates of viral isolation [32]; third, we replaced the requirement for time-intensive epifluorescent microscopy with identification of putative viral infection by comparing infected and uninfected hosts by flow cytometry, followed by confirmation using transmission electron microscopy (TEM). These clades are abundant and important to global carbon biogeochemistry [23, 33–37], but little is known about their associated viruses. In the case of viruses infecting SAR11, two challenges limit our ability to evaluate host–virus ecology in natural communities: (1) Assembly of abundant and microdiverse genomes from viral metagenomes presents a challenge to short-read assembly methods, resulting in underrepresentation in subsequent datasets [12, 18]. This was demonstrated in the successful isolation of the first known pelagiphages by culturing, including the globally dominant HTVC010P, which, prior to its isolation, was entirely missed in marine viromes [38]; (2) poor representation of viral taxa in databases limits our capacity to accurately train machine-learning approaches for in silico host prediction [12, 39, 40]. This results in either a lack of host information for abundant viral contigs, or worse, incorrect assignment of host to viral contigs, confounding ecological interpretation of data. Some important taxa, such as the OM43 clade, which plays an important role in oxidation of volatile carbon associated with phytoplankton blooms [33, 35, 41], lack any isolated viruses with experimentally confirmed hosts. In addition, both SAR11 and OM43 represent model organisms for genome streamlining as a result of nutrient-limited selection [42]. The effect of genome minimalism on viral infection dynamics is poorly understood, but critical to evaluating the impact of predator-prey dynamics on global marine carbon budgets. In this study, novel SAR11 and OM43 representatives from the Western English Channel were isolated and used as bait to isolate associated viruses. We increased the initial concentration of viruses in natural seawater samples by tangential flow filtration, followed by inoculation of cultures and one to three rounds of sequential enrichment on target hosts in 96-well plates. This yielded 117 viral isolates from 218 inoculated cultures from seven monthly water samples (September 2018–July 2019). A subsample of putative viral isolates for both clades was sequenced, providing 13 novel viral genomes, including the first known siphovirus to infect SAR11 and the first known virus–host model for OM43.

## Results and discussion

### Isolation of a novel SAR11 strain and three new OM43 strains from the Western English Channel to use as bait for phage isolation

Dilution-to-extinction culturing for host taxa using natural seawater-based medium was performed from a water sample collected in September 2017 from the Western English Channel and yielded the first SAR11 strain (named H2P3α) and the first three OM43 strains (named C6P1, D12P1 and H5P1) from this region. The full-length 16S rRNA gene of *Pelagibacter sp*. H2P3α was 100% identical to that of the warm-water SAR11 ecotype *Pelagibacter bermudensis* HTCC7211 (subclade 1a.3) and was considered to be a local variant [43] (Supplementary Fig. 1A). All three novel OM43 isolates were most closely related to *Methylophilales sp*. HTCC2181, a streamlined member of the OM43 clade with a 1.3-Mbp genome, isolated from surface water of the North Eastern Pacific [44] (C6P1 96.17%, D12P1 96.62% and H5P1 97.79% nucleotide identity across the full 16S rRNA gene) (Supplementary Fig. 1B). The average nucleotide identity of the 16S rRNA gene of isolates CP61, D12P1 and H5P1 to each other was ~98.46% (Supplementary Table 1), suggesting that they are representatives of the same genus [45].

### An efficient, low-cost method of isolating new viruses yielded 117 new viral isolates for SAR11 and OM43 taxa

**Fig. 1:**
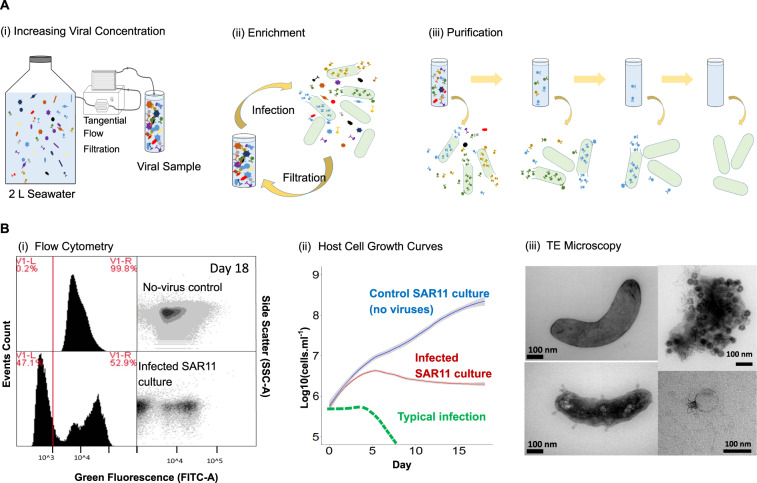
Schematic of the isolation workflow. **A** Isolating and purifying viral cultures by: (i) Increasing concentration of viruses in water samples by tangential flow filtration (TFF); (ii) Initial infection of host cultures to enrich the sample for specific viruses; (iii) Purification of viral isolates through three rounds of dilution-to-extinction. **B** Screening cultures for viral infections using: (i). Flow cytometry, by comparing populations of no-virus controls and infected cultures; (ii) Comparing growth curves of no-virus control culture (HTCC1062) against infected SAR11 cultures; (iii) Confirming the presence of viruses in infected SAR11 cultures using transmission electron microscopy: Top left: HTCC1062 no-virus control, bottom left: infected HTCC1062, top right: aggregated cellular debris and viruses, bottom right: virus found in infected HTCC1062 culture.

**Fig. 2:**
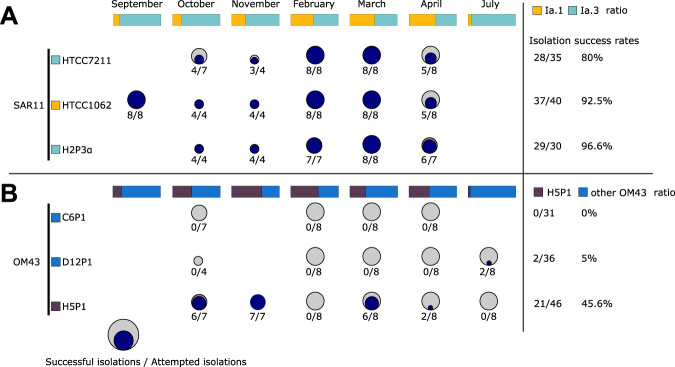
Summary of success rates of viral isolations per bacterial host. **A** Isolation rates for phages on three SAR11 strains HTCC1062, HTCC7211 and H2P3α; and **B** Isolation rates for phages on three OM43 strains C6P1, D12P1 and H5P1. Circles and associated numbers represent the number of successful isolations (i.e. successful passage through three rounds of dilution-to-extinction), over the total number of attempts made. Bars at the top of each panel represent the relative abundance (SAR11 subclades 1a.1 and 1a.3, and OM43 strains for **A** and **B**, respectively) of strains in the microbial community sampled at the time of collection (based on 16S rRNA community profiling). Percentages on the right represent total successes for all attempts per host strain.

Using the four new hosts from above and established SAR11 isolates *Pelagibacter ubique* HTCC1062 (subclade 1a.1) and HTCC7211 (subclade 1a.3), we developed an optimised viral isolation pipeline (Fig. 1) and applied it to 6 monthly water samples from the Western English Channel, taken between September 2018 and April 2019 (Supplementary Table 2). Each month, we concentrated a natural viral community with tangential flow filtration and used it to inoculate one to two 96-well Teflon plates containing host cultures at ~10^6^ cells mL^−1^. Plates were monitored by flow cytometry and growth of putatively infected cultures was compared to those of unamended controls over the course of ~2 weeks to account for the slow growth rates of SAR11 and OM43 [35, 46]. An additional sample was taken in July 2019 to attempt viral isolation on OM43 strains D12P1 and H5P1. Out of a total of 218 cultures amended with concentrated viral populations, 117 viruses were isolated, purified and still infective after at least three passages, with repeatable differences observed in cytograms of infected and control cultures (Supplementary Figs. 2–5). This represents an overall isolation efficiency of 53% and an average yield of 18 viruses per environmental sample (Fig. 2). For 90% of inoculated SAR11 cultures (94 out of 105), we observed evidence of viral infection, and the putative viral isolate could be propagated and purified, fulfilling Koch’s postulates for confirming a pathogenic agent. For OM43, 23 out of 113 (20%) inoculated cultures yielded positive infections that could be similarly propagated. All viral isolations required between one and three rounds of virus enrichment (Supplementary Table 3) before changes in host growth curves between infected and uninfected cultures could be observed. This suggests that putatively rare viruses can be enriched within three rounds to a level at which infection can be observed on a flow cytometer.

### New viruses represent novel viral populations and support established ANI cut-offs for ecologically discrete viral ecotypes

Due to the rate-limiting step of culturing sufficient biomass for extraction of viral DNA, we subselected 16 viral isolates based on availability in November 2018 across four different hosts (HTCC1062, HTCC7211, H2P3α and H5P1) for Illumina sequencing to >30-fold coverage (Table 1). Three out of 16 sequenced samples (~19%, two from host HTCC7211, one from OM43 host H5P1) failed to assemble into single viral contigs, in line with previously reported failure rates of 18–39% for phages of *Escherichia coli* and *Salmonella spp*. [47]. For 11 of the remaining 13 samples (12 from SAR11 hosts and one from OM43), each individual sequence assembly was identified as a complete viral genome by VirSorter [15] and 95–100% complete using CheckV [48]. All assemblies yielded a single viral contig (categories 1 or 2, >15 kbp) per sample, indicating that our purification process was effective in recovering pure viral isolates. Interestingly, all viral isolate genomes were classified by VirSorter as Category 2 (“likely virus”) rather than Category 1 (“most confident”), despite being complete—indicating either a lack of viral hallmark genes or a lack of enrichment of viral genes on the contigs. This finding matches our own observations for other isolated viruses infecting SAR11 (data not shown) and suggests that VirSorter classification of pelagiphages is conservative. Viral isolates were named in accordance with current ICTV guidelines for prokaryotic virus taxonomy [49], with names selected from Norse mythology and folklore, and contemporary culture (Table 1).

**Table 1:** Summary of phages isolated and sequenced in this study.

Viral collection number	Virus	Host	Viral population representative	Hypergeometric cluster	Genome	Length (bp)	G + C %	Taxon	Accession number	Simplified phonetic spelling	Meaning and origin of the names
EXVC025P	Eistla	HTCC1062	Eistla	Cluster A	Circular	39638	32.7	Podoviridae	MT375521	ais:tla:	“Stormy one”, Giantess in the poetic Edda
EXVC018P	Eyrgjafa	HTCC1062	Eyrgjafa	Cluster B	Circular	38005	32.6	Podoviridae	MT375523	e:ɪrgja:fa:	“Scar donor”, Giantess in the poetic Edda
EXVC020P	Gjalp	HTCC1062	Eyrgjafa	Cluster B	Circular	37857	32.5	Podoviridae	MT375524	gja:lp	“Roaring one”, Giantess in the poetic Edda
EXVC021P	Greip	HTCC1062	Greip	Cluster D	Circular	34916	31.5	Podoviridae	MT375525	graip	“Grasp”, Giantess in the poetic Edda
EXVC014P	Ran	H2P3α	Ran	Cluster A	Circular	41529	34.1	Podoviridae	MT375530	ran	“Plundering”, Goddess of the Sea
EXVC013S	Aegir	H2P3α	Kolga	Singleton	Fragment	18297	31.1	Siphoviridae	MT375519	æ:ɡir	“Sea”, God of the Sea
EXVC012P	Jörmungand	H2P3α	Ran	Cluster A	Circular	41529	34.1	Podoviridae	MT375528	jɔrmunˌɡand	“Huge monster”, Giant sea serpent
EXVC016S	Kólga	H2P3α	Kolga	Singleton	Circular	48659	30.5	Siphoviridae	MT375529	kɔlga:	“Cool-wave”, Daughter of Ran and Aegir
EXVC019P	Unn	H2P3α	Bylgja	Cluster A	Circular	41069	33.5	Podoviridae	MT375531	ʊ:n	“Wave”, Daughter of Ran and Aegir
EXVC015P	Hroenn	H2P3α	Bylgja	Cluster A	Circular	41069	33.5	Podoviridae	MT375527	ɦrøn	“Wave”, Daughter of Ran and Aegir
EXVC010P	Bylgja	H2P3α	Bylgja	Cluster A	Circular	41069	33.5	Podoviridae	MT375520	bɪ:lgja:	“Billow”-wave, Daughter of Ran and Aegir
EXVC011P	Himinglæva	H2P3α	Bylgja	Cluster A	Circular	41069	33.5	Podoviridae	MT375526	hi:mɪŋɠlæfa	“Transparent-on-top”-wave, Daughter of Ran and Aegir
EXVC282S	Venkman	H5P1	Venkman	Singleton	Linear	38624	34.4	Siphoviridae	MT375522	vɛŋkmæn	Bill Murray’s Character in the Ghostbusters movie

Viral populations are defined as discrete ecological and evolutionary units that form non-overlapping clouds of sequence space, and previous work in cyanophage populations has shown that viral populations can be delineated into populations using an average nucleotide identity (ANI) cutoff of 95% [50]. Pairwise ANI was calculated between the thirteen successfully sequenced viral genomes from this study and 85 other known or putative pelagiphages [39, 51, 52]. Pairwise ANI ranged between 77.5 and 100%, with a discrete distribution between 96.4 and 100.0% (Supplementary Fig. 6, Supplementary Table 4). This is in agreement with previous work in cyanophages [50, 53] and supports the broad use of proposed boundary cut-offs to define viral populations within viral metagenomic assemblies [10, 11]. At the proposed ANI cut-offs of 95% over 85% length [18], our 13 new viruses clustered into six viral populations, ranging from singletons to a viral population with four members (Table 1). Phages within the same populations were all isolated from the same environmental sample and on the same host, in agreement with their classification as discrete ecological and evolutionary units. All viruses isolated in this study formed their own exclusive viral populations, with no representatives from either known isolates [38, 52] or fosmid-derived [39] genomes from other studies, indicating that a high degree of viral population diversity remains to be discovered in the Western English Channel and beyond.

### Current pelagiphage isolates can be organised into five distinct phylogenetic clades

**Fig. 3:**
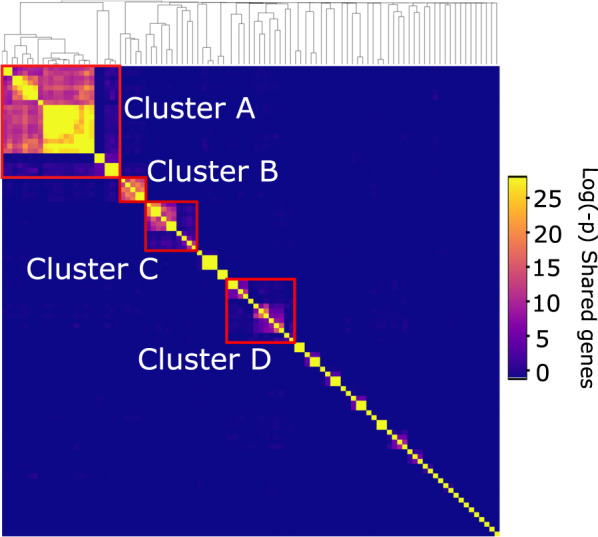
Pelagibacter podophages can be grouped into four main clusters. Hypergeometric probability of shared-gene content between known pelagiphage genomes identified four main viral phylogenetic clusters (outlined with red boxes). Siphophage Kolga and myophage HTVC008M did not cluster with podophages based on shared gene content (singletons). Cluster C only contained metagenomically derived contigs. Cluster assignment for phages isolated in this study can be found in Table 1. See Supplementary Fig. 7 for a full phylogenetic tree.

To evaluate isolate diversity at higher taxonomic organisation, we picked one representative from each of our six viral populations and compared them to previous isolates and fosmid-derived phage sequences using three approaches: first, phylogenetic analyses were performed based on conserved genes in known pelagiphage isolates and closely related taxa from viral metagenomic surveys [11, 12]; second, raw hypergeometric probability of shared-gene content was calculated (to capture broader relationships and account for genomic mosaicism) [54]; third, genomes were organised into ICTV-recognised genera using vConTACT2. vConTACT2 initially derives viral clusters using a hypergeometric approach, with subsequent refinement with Euclidean-distance-based hierarchical clustering to split mismatched, ‘lumped’ viral clusters [19]. All three approaches were congruent—clustering on probability of shared-gene content organised pelagiphage genomes into four main clusters and numerous singleton genomes (Fig. 3). This was broadly supported by phylogenetic (Supplementary Fig. 7) and vConTACT2 (Supplementary Fig. 8) classification approaches. Cluster A contained 23 members (nine from fosmid-derived contigs [39]; eleven previously isolated pelagiphages [52, 55]) and Pelagibacter phages *Ran* (EXVC014P), *Bylgja* (EXVC010P) and *Eistla* (EXVC025P) from this study. Cluster B contained two previously isolated pelagiphages, one fosmid-derived contig and Pelagibacter phage *Eyrgjafa* (EXVC018P) from this study. All viruses in Clusters A and B were assigned to a single viral genus by vConTACT2 that also contained 12 previously isolated pelagiphages [38, 52]. Cluster C only contained fosmid-derived contigs from the Mediterranean [39], with no isolated representatives, marking it an important target for future isolation attempts. Cluster D contained eight fosmid-derived contigs, Pelagibacter phage HTVC010P and Pelagibacter phage *Greip* (EXVC021P) from this study. Pelagibacter phage *Kolga* (EXVC016S) from this study and the only known Pelagimyophage HTVC008M from a previous study [38] fell outside the four main clusters. VConTACT2 split Cluster D, leaving Pelagibacter phages *Greip* and *Kolga* as members of two singleton clusters, suggesting that they are the first cultured representatives of novel viral genera and distinct from the globally ubiquitous Pelagibacter phage HTVC010P.

### Novel pelagiphages are ecotype-specific and persist in the community

Community composition analysis using 16S rRNA genes showed that during the sampling period (September 2018–July 2019), the SAR11 contribution relative to the total number of sequences ranged from a minimum of 28.0% in July 2019 to a maximum of 42.2% in September 2018 (Supplementary Fig. 9). At the clade level, SAR11 composition was relatively stable over time, except for clade II roughly doubling its relative contribution from 15.1% to about 29.6% between February and April. The SAR11 community was dominated by clade I overall throughout the sampling period. Within clade I, the ratio between SAR11 subclade Ia.1 (cold-water ecotype) and Ia.3 (warm-water ecotype) showed that the warm-water ecotype dominated from September to November as well as July (Fig. 2A). During the coldest months in the Western English Channel (February–April), the cold-water ecotype became as abundant as the warm-water ecotype with roughly a 1:1 ratio. Overall, temperature ranged from 9.3 °C to 14.1 °C from October to April, when isolations were attempted on all strains (Supplementary Table 2). HTCC1062 and HTCC7211 have specific growth rates of ~0.22 and ~0.12 divisions per day at 10 °C, respectively [46]. Our new SAR11 isolate from the Western English Channel, *Pelagibacter sp*. H2P3α, showed similar growth rates to *Pelagibacter bermudensis* HTCC7211 (Supplementary Fig. 10), with specific growth rates of ~0.10, ~0.45 and ~0.84 divisions per day at 10, 18 and 25 °C, respectively. Therefore, measured in situ temperatures during our sampling period were sufficient to support slow growth of warm-water ecotypes even during winter months, potentially providing sufficient prey to support a population of warm-water ecotype-specific phages. This result shows that slow growth of warm-water ecotypes at in situ temperatures is possible, supporting the finding of the 16S community analysis that this ecotype persists throughout the year.

An alternative explanation is that isolated viruses have a broad host range that encompasses both warm- and cold-water ecotypes of the SAR11 subclade Ia. We tested the host range (Table 2) of six pelagiphages (one of each viral population), isolated from samples in October 2018 and November 2018 (in situ water temperature of 14.8 °C and 14.2 °C, respectively) across the three SAR11 strains (Table 1). Pelagibacter phages *Eistla, Eyrgjafa* and *Greip* all infected cold-water ecotype HTCC1062 exclusively, while Pelagibacter phages *Ran* and *Kolga* only infected warm-water ecotypes HTCC7211 and H2P3α. Pelagibacter phage *Bylgia* was the only virus that could infect both warm- and cold-water ecotypes. Therefore, our new pelagiphages appear to be broadly ecotype-specific, confirming previous findings [52]. Our results suggest overall that pelagiphages persist in the water column throughout the year in sufficient densities to be isolated by our enrichment method, despite ecotypic specificity and fluctuations in warm- and cold-water ecotype community contributions of SAR11 subclade 1a. If concentration and enrichment of viruses during isolation is sufficient to successfully isolate even low-abundance phages, then a comprehensive library of representative phage isolates could be generated with relatively modest sampling effort across a few locations.

**Table 2:** Host infectivity of viral populations isolated and sequenced in this study.

Phage	H2P3α	HTCC7211	HTCC1062
Eistla			+
Eyrgjafa			+
Greip			+
Ran	+	+	
Kolga	+	+	
Bylgja	+	+	+

*Pelagibacter sp.* H2P3α and *Pelagibacter bermudensis* HTCC7211 are warm-water ecotypes of SAR11 subclade 1a; HTCC1062 is a cold-water ecotype*. Pelagibacter* phage *Bylgja* was the only virus capable of infecting both ecotypes.

### Pelagibacter phages Kolga and Aegir—the first siphovirus infecting SAR11

**Fig. 4:**
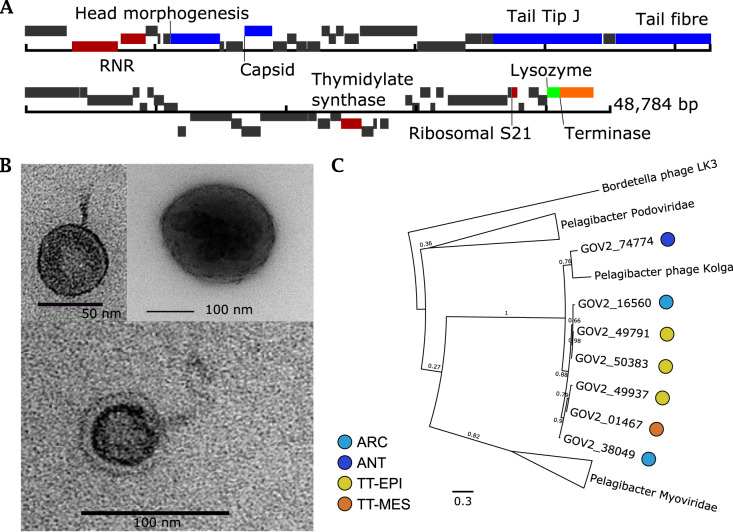
The first reported siphovirus infecting SAR11 (*Pelagibacter* phage *Kolga* EXVC016S) isolated on novel host *Pelagibacter sp*. H2P3α. **A** Gene map of the 48,784 bp genome, which contains 80% hypothetical genes without known function. **B** Transmission electron micrographs of *Kolga* (left and bottom) and an H2P3α host cell infected with *Kolga* (top right). **C** Unrooted maximum likelihood tree of *terL* genes found in pelagiphages. Branches containing members of the Podoviridae and Myoviridae family infecting SAR11 are collapsed for clarity (full tree is available in Supplementary Fig. 7). Closely related contigs prefixed with ‘GOV’ represent viral contigs from the Global Ocean Virome dataset and the ecological zone from which they were assembled is marked: temperate–tropical epipelagic (TT-EPI), temperate–tropical mesopelagic (TT-MES), Antarctic (ANT), Arctic Ocean (ARC).

The 25 previously known viral isolates infecting SAR11 comprise 24 podoviruses and one myovirus [38, 52, 56]. Previous cultivation efforts for viruses of SAR11 have not isolated any siphoviruses, nor are any known from viral metagenomic studies. We isolated and sequenced the two Pelagibacter phages *Kolga* and *Aegir* using host H2P3α as bait (Fig. 4A-C). Transmission electron microscopy (TEM), which showed evidence of a long tail (Fig. 4B), suggested classification as the first reported siphovirus infecting members of the SAR11 clade.

Pelagibacter phages *Aegir* and *Kolga* were classified as members of the same population using a boundary cutoff of 95% ANI over 85% contig length; however, *Aegir* had a length of 18,297 bp compared to 48,659 bp in *Kolga*; therefore, we considered *Aegir* to be a partial genome of the same viral population. *Kolga* did not share a significant number of genes with known SAR11 podoviruses (Fig. 3), and did not cluster with other pelagiphages using hypergeometric analysis based on shared-gene content. VConTACT2 also grouped *Aegir* and *Kolga* into one cluster without any other known viruses, suggesting that it represents a novel viral genus. A number of genes found in *Kolga* were shared with other known siphoviruses (associated with different hosts) such as the Bordetella phage LK3. Screening of contigs from the Global Ocean Virome dataset [11] identified six contigs from various ecological zones, which shared a viral cluster with *Kolga*, but which belonged to different viral populations based on network analysis using vConTACT2. Phylogenetic analysis of all Pelagibacter phage TerL genes indicates that the closest known relatives to *Kolga* and *Kolga*-like contigs are members of the Myoviridae family, supporting a classification as a distinct and novel viral group (Fig. 4C, Supplementary Fig. 7). In *Kolga*, 67% of encoded genes could not be functionally annotated, and out of all hypothetical genes identified on *Kolga*, only three hypothetical genes were shared with SAR11 podoviruses. In contrast, on average, ~90% of genes without known function identified within our novel SAR11 podoviruses are shared between different SAR11 podoviruses. *Kolga* possesses a tail- tip J protein (Fig. 4A), often found in phages with long non-contractile tails such as E. coli phage ƛ, where it plays a role in DNA injection during cell entry and tail assembly [57]. *Kolga* also encodes a small S21 subunit of the 30S ribosomal gene structurally similar to the ones found in Pelagibacter phage HTVC008M, Pelagimyophage-like contigs [58] and hosts HTCC7211 and H2P3α. Encoding ribosomal genes is a feature found in numerous myoviruses and siphoviruses [59]. The S21 gene is involved in translation initiation and needed for the binding of mRNA [60]. Virally encoded S21 genes may provide a competitive advantage for the phage as it could replace cellular S21 and assist in the translation of viral transcripts. *Kolga* may need its S21 gene for shifting the translational frame, as it has been shown that for some members of the Caudovirales, the production of tail components is dependent on programmed translational frameshifting [61]. Given the constitutive nature of gene expression in genomically streamlined bacteria [62], genes such as S21 may also provide the virus with a mechanism to manipulate host metabolism in the absence of typical promoters and repressors.

### First methylophages for marine OM43 isolated

Isolation of novel phages for the OM43 clade yielded 23 positive infections, with efficiencies ranging from 0% (no viruses isolated on host C6P1) to 45% on H5P1 (Fig. 2). To the best of our knowledge, these are the first reported viruses infecting members of the OM43 clade. One explanation for the lower efficiency of isolation of phages infecting OM43 is simply one of lower host abundance concomitant with lower phage abundance in the viral community, reducing the likelihood of infective viruses coming into contact with susceptible and permissive cells. OM43 is closely associated with metabolism of extracellular substrates from phytoplankton blooms [63], but has low abundance outside of phytoplankton spring blooms [33]. Our water samples were not associated with high in situ fluorescence (used as proxy measurement for phytoplankton), and missed the April 2019 spring bloom by about two weeks (Supplementary Fig. 11). Based on 16S community analysis, the OM43 contribution to the bacterial community (0.7%) was the lowest during sampling in April 2019 and highest in October 2018 (1.9%). Relative to all other OM43, H5P1 was the most abundant OM43 in the Western English Channel, contributing more than half of all OM43 in November (Fig. 2B). This could explain the higher success rate of isolating phages on host H5P1 compared to C6P1 and D12P1.

**Fig. 5:**
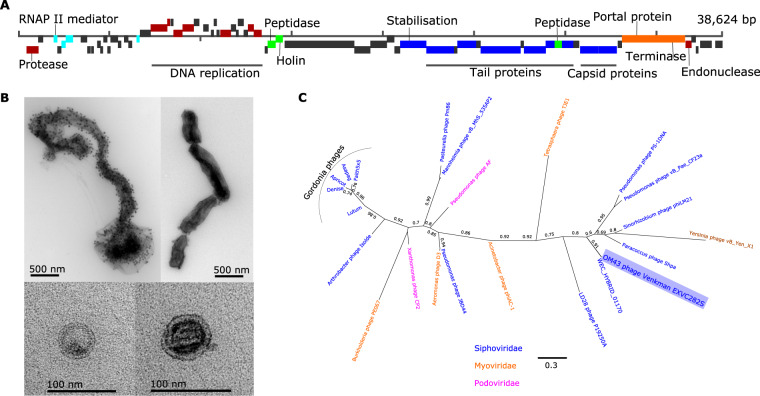
The first reported cultured virus known to infect a member of the OM43 clade (Methylophilales phage Venkman EXVC282S), which was isolated on host strain H5P1 from this study. **A** Gene map displaying protein-coding genes. Gene function is colour-coded as follows: structural genes (blue); DNA replication (red); lysis (green); transcription (turquoise); packaging (orange); hypothetical genes (grey). **B** TEM images of infected and chaining H5P1 cells (top left), uninfected H5P1 chaining cells (top right), *Venkman* viral particles (bottom left and right). **C** Maximum likelihood tree (500 bootstraps) of concatenated viral *terL* and exonuclease genes. Host families of the phages are indicated in the figure.

Sequencing of the first OM43 phage isolate, Methylophilales phage *Venkman* (EXVC282S), returned a single genome 38,624 bp long (31.9% GC content), which was linear but complete (Supplementary Table 5). *Venkman* encodes genes (Fig. 5A) with similar synteny and function to the siphovirus P19250A (38,562 bp) that infects freshwater *Methylophilales* LD28, which is often considered a freshwater variant of OM43 [64, 65]. Unlike the siphovirus P19250A, TEM images indicated that OM43 phage *Venkman* had a short tail (Fig. 5B) similar to podoviruses, though it is possible that tail structures were lost during grid preparation. *Venkman* shared a viral population with a 23-kbp contig from the Western English Channel virome assembled from short-read data (WEC_HYBRID_01170), suggesting that this viral type does not suffer from the issues of high abundance and microdiversity that challenge assembly of some pelagiphages. Phylogenetic analysis of concatenated TerL and exonuclease genes indicated that *Venkman* is most closely related to P19250A and other siphoviruses infecting different Proteobacteria (Fig. 5C). However, branch support values were low, despite numerous attempts to refine the tree with different approaches (see Supplementary Methods). We identified a number of common phage proteins such as a capsid protein, terminase, nucleases and tail structural proteins, and the remaining 54% of genes were hypothetical. VConTACT2 assigned OM43 phage *Venkman* and LD28 phage P19250A to the same genus-level cluster; therefore, the OM43 phage *Venkman* may be a marine variant of the freshwater LD28 siphovirus P19250A.

### Global abundance of novel isolates highlights niche specificity and low representation in existing datasets

**Fig. 6:**
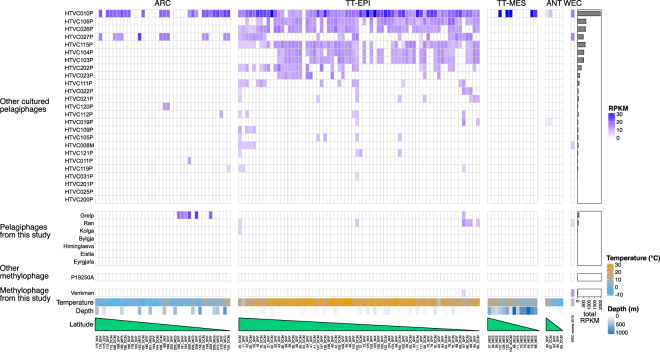
Relative abundance (reads recruited per kilobase of contig per million reads (RPKM)) of known pelagiphage isolates and methylophage genomes in viromes from the Western English Channel [12] and the Global Ocean Virome [11]. Samples are organised by decreasing latitude per ecological zone: ARC Arctic, TT-EPI temperate–tropical epipelagic, TT-MES temperate–tropical mesopelagic, ANT Antarctic, WEC Western English Channel.

To evaluate the relative global abundance of our new phage isolates, existing virome datasets from the Global Ocean Virome survey [11] and the Western English Channel [12] were randomly subsampled to 5 million reads and competitively recruited against genomes of viral population representatives from this study as well as previously isolated pelagiphages (see Supplementary methods). Overall, with the exception of Pelagibacter phage HTVC010P, pelagiphages were poorly represented in samples from temperate–tropical mesopelagic (TT-MES) and Antarctic (ANT) ecological zones, as defined in [11] (Fig. 6). Phages isolated in this study were neither globally ubiquitous, nor abundant in the single Western English Channel virome (with the exception of phage *Ran*). Phages *Bylgja, Himinglaeva, Eistla* and *Eyrgjafa* did not achieve the minimum cutoff of 40% genome coverage to be classified as present [18] in any of the viromes tested. *Ran* was the only phage isolated in this study with representation in at least two samples from temperate–tropical epipelagic (TT-EPI) zones, concordant with its host specificity of warm-water SAR11 ecotypes (*Pelagibacter bermudensis* HTCC7211 and H2P3α) from this study (Table 2). Similarly, the Western English Channel virome was taken in September 2016, when waters are usually highly stratified after summer heating [66] and warm-water ecotypes of SAR11 dominate the microbial community (Fig. 2A). Other viral populations from this study also isolated on warm-water SAR11 ecotypes (phages *Kolga, Bylgja* and *Himinglaeva*) were either absent or below limits of detection in this sample. This coupled with the low global abundance of these viruses suggests that pelagiphage communities comprise few highly abundant taxa and a long rare tail. Pelagibacter phage *Greip* was detected in seven samples, six of which were Arctic (ARC) samples from a discrete region of the Arctic characterised by low nutrient ratios [11]. In three of those samples (191_SRF, 193_SRF and 196_SRF), HTVC010P was not detected and in the other three (206_SRF, 208_SRF and 209_SRF), *Greip* was 1.5- to 6.6-fold more abundant than HTVC010P, identifying *Greip* as an abundant arctic pelagiphage ecotype. The fact that *Greip* was isolated on the cold-water ecotype of SAR11 (Pelagibacter ubique HTCC1062) and does not infect either warm-water ecotype HTCC7211 or H2P3 supports the hypothesis that host niche specificity shapes the phylogeography of associated viral taxa.

In the Western English Channel viral metagenomes, OM43 phage *Venkman* was the third most abundant (2,699 RPKM) after Pelagibacter phages HTVC027P (5,023 RPKM) and HTVC010P (5,026 RPKM) identifying it as an ecologically important virus in this coastal microbial community (Fig. 6). In contrast, neither *Venkman* nor LD28 phage P19250A were identified in the majority of samples from GOV2, except in one sample (67_SRF) in the South Atlantic Ocean (257 RPKM against *Venkman*). Sample 67_SRF was also classified as a coastal biome by the authors [11]. These results coincide with previous reports that OM43, and presumably their associated viruses as a consequence of host abundance, are important in some coastal regions, but largely absent in open-ocean systems [33, 41].

### Unusual host–virus dynamics are prevalent in isolated phages

Pelagibacter phages *Bylgja*, *Eyrgjafa*, *Ran* and *Eistla* all encode endonucleases and exonucleases (Supplementary Fig. 12) and cluster by shared protein content with other pelagiphages (Clusters A and B), such as, e.g. HTVC011P and HTVC025P, shown previously to integrate into host genomes (Fig. 3) [52]. We therefore predict that all our phages within Clusters A and B are temperate phages. *Eyrgjafa* also encodes a tRNA-Leu that has 85% nucleotide identity over the first 34 bases of the tRNA-Leu of its host HTCC1062, suggesting a putative integration site into the host genome [67]. To date, 16 of the 29 viruses previously isolated on SAR11 strains have either been shown to be capable of lysogeny, and/or encode genes associated with a temperate infection cycle. In contrast, viruses such as the Pelagibacter phage *Greip*, and the abundant HTVC010P in Cluster D, do not possess any genes associated with lysogeny, and would therefore be classified as exclusively lytic. Viruses in Clusters A and B (putatively temperate phage), were of much lower abundance in the environment compared with HTVC010P, which was among the most abundant pelagiphages in the Western English Channel (Fig. 6), suggesting a possible ecological difference between two groups of viruses with different infectivity strategies.

Interestingly, growth curves of hosts infected with Pelagibacter phage *Greip* and other isolated phages deviated from the expected decay in cell abundance associated with viral lysis and previously observed in isolated pelagiphages [38] (Fig. 1 Bii). The first pelagiphages HTVC010P and HTVC008M were isolated from the warm waters of the Sargasso Sea, and HTVC011P and HTVC019P were isolated from the colder waters of the Oregon Coast. All four strains were propagated on the cold-water SAR11 ecotype *P. ubique* HTCC1062. In all cases, host density was reduced from ~8 × 10^6^ cells mL^−1^ at T_0_ to <10^6^ cells mL^−1^ over a 60–72 h period. Viruses isolated from warm waters took 17% longer than those from cold water to do so [38], suggesting that suboptimal hosts reduced the rate of infection as shown in cyanophages [68]. In contrast, infection dynamics of our isolates often resulted in host density of infected cultures growing to a steady state, but at a lower cell density than uninfected cells (Supplementary Video 1), irrespective of cluster, population assignment or evidence of genes associated with temperate lifestyles. Out of 117 viruses isolated in this study, only 16 infections reduced host abundance below their inoculum density of 10^6^ cells mL^−1^. In 53 infections, densities of infected cells increased to within an order of magnitude of uninfected cells (Supplementary Fig. 13), but demonstrated clear evidence of viral infection in cytograms, TEMs and subsequent recovery of viral genomes in selected samples.

Similar patterns of infection were recently reported in the extremely abundant bacteriophage ɸCrAss001 found in the human gut, where infection of exponentially growing cells of *B. intestinalis* 919/174 did not result in complete culture lysis, but caused a delay in stationary phase onset time and final density, despite lacking genes associated with lysogeny. As with our study, the authors observed that this only occurred in liquid culture, and isolation of the virus required numerous rounds of enrichment. They postulated that the virus may cause a successful infection in only a subset of host cells, with the remainder exhibiting alternative interactions such as pseudolysogeny or dormancy [32]. The prevalence of similar infection dynamics in the phages isolated in this study offers two intriguing possibilities: (i) many of the viruses isolated in this study are either not fully lytic, but fall somewhere on the continuum of persistence [69]. This could be controlled by genes currently lacking a known function, with lysogeny (and associated superinfection immunity) favoured at high cell density, which would support the Piggyback-the-Winner hypothesis [70, 71]; (ii) the steady state of host and virus densities observed here is an indicator of host phenotypic bistability in these streamlined heterotrophic taxa. Viral propagation occurring in only a subset of cells could explain the requirement of multiple rounds of enrichment before sufficient viral load is reached to be able to observe lytic infection on the host population. Either strategy, or a combination of both, would provide an ecological advantage of long-term stable coexistence between viruses and hosts, and offers an explanation of the paradox of stable high abundances of both predator and prey across global oceans [12, 38, 72, 73]. Infection in a subset of the population could also explain the low lytic activity observed in pelagiphages in situ, despite high host densities [74], and the small dynamic range and decoupled abundances of SAR11 and virioplankton in the Sargasso Sea [75]. Limited lysis of subpopulations of hosts such as SAR11 and OM43 that specialise in the uptake of labile carbon enriched through viral predation [76, 77] could facilitate efficient intra-population carbon recycling and explain the limited influence of SAR11 and associated viral abundances on carbon export to the deep ocean [5]. We propose the moniker the ‘Soylent Green Hypothesis’ for this mechanism, after the 1973 cult film in which the dead are recycled into food for the living. Further investigation leveraging our new virus–host model will provide greater insight into viral influence on ocean carbon biogeochemistry.

In conclusion, our method coupled dilution-to-extinction cultivation of hosts and associated viruses, resulting in the isolation of three new strains of OM43: a Western English Channel variant of a warm-water ecotype of SAR11, the first known methylophages for OM43 and the first siphovirus infecting SAR11, as well as eleven other viruses infecting this important marine heterotrophic clade and >100 more isolates to be sequenced and explored. The described method represents an efficient and cost-effective strategy to isolate novel virus–host systems for experimental evaluation of co-evolutionary dynamics of important fastidious taxa from marine and other biomes. Coupling these methods to existing advances in host cultivation requires minimal additional effort and will provide valuable representative genomes to improve success rates of assigning putative hosts to metagenomically derived viral contigs. Broader representation of model systems beyond cyanophages and viruses of copiotrophic, *r-*strategist hosts will reduce bias in developing methods to delineate viral population boundaries [78, 79], increasing the accuracy with which we derive ecological meaning from viral metagenomic data. We therefore hope that this method will enable viruses to be included in the current resurgence of cultivation efforts to better understand the biology and ecology of phages, and the influence of the world’s smallest predators on global biogeochemistry.

## Methods summary

A complete description of the materials and methods is provided in the Supplementary Information. Four bacterial strains (*Methylophilales sp*. C6P1, D12P1 and H5P1; *Pelagibacter sp*. H2P3α) were isolated from Western English Channel station L4 seawater samples using dilution-to-extinction methods [29]. All four bacteria and two additional SAR11 strains *Pelagibacter bermudensis* HTCC7211 and *Pelagibacter ubique* HTCC1062 were used as bait to isolate phages from six monthly Western English Channel L4 seawater samples (50°15.00 N; 4°13.00 W). Briefly, water samples were concentrated for viruses using tangential flow filtration (100 kDa Hydrosart membrane) and used as viral inoculum (10% v/v) in exponentially growing cultures of host bacteria in artificial seawater medium [80] in 96-well Teflon plates (Radleys, UK). Cells of the resulting lysate were filtered out (0.1 µm PVDF syringe filters) and the filtrate was used as viral inoculum in another round of isolation. This process was repeated until viral infection could be detected by flow cytometry—comparing cytograms and maximum density of infected cultures against uninfected cultures. Phages were purified by dilution-to-extinction methods (detailed protocol available here: 10.17504/protocols.io.c36yrd). Phage genomes were sequenced using Illumina 2 × 150 PE sequencing, assembled and manually annotated as described in [81]. Phylogenetic classification of phages was performed on concatenated shared genes using a combination of Bayesian inference trees, maximum likelihood trees and shared-gene likelihood analyses, depending on the availability of appropriate taxa. ICTV-recognised genera based on shared-gene content were assigned with VConTACT2 [19]. The relative abundance of novel phages in the Western English Channel [12] and Global Ocean viromes [11] was estimated (RPKM) by competitive read recruitment of five million randomly subsampled reads against pelagiphage and methylophage genomes from this study and others [38, 52, 64, 82].

## Supplementary information

Supplementary Information

Supplementary Figure 1

Supplementary Figure 2

Supplementary Figure 3

Supplementary Figure 4

Supplementary Figure 5

Supplementary Figure 6

Supplementary Figure 7

Supplementary Figure 8

Supplementary Figure 9

Supplementary Figure 10

Supplementary Figure 11

Supplementary Figure 12

Supplementary Figure 13

Supplementary Table 1

Supplementary Table 2

Supplementary Table 3

Supplementary Table 4

Supplementary Table 5

Supplementary Video 1

## Data Availability

All reads can be found in the SRA database under BioProject number PRJNA625644 as BioSamples SAMN14604128–SAMN14604140. Annotated phage genomes are deposited as GenBank submissions under accession numbers MT375519–MT375531. Sequences used for phylogenetic analysis are deposited under https://github.com/ViralPirates/viral-dte.
