# Supplementary material for: Efficient dilution-to-extinction isolation of novel virus–host model systems for fastidious heterotrophic bacteria
Source: ISME J. 2021 Jan 25;15(6):1585–98. doi: 10.1038/s41396-020-00872-z (PMC8163748; doi:10.1038/s41396-020-00872-z)
Supplement: Supplementary file 1 — Supplementary Information [file 41396_2020_872_MOESM1_ESM.docx]

**Supplementary Information**

**Supporting Methods**

*Water sampling*: A total of 20 L of seawater was collected in rosette-mounted Niskin bottles at a depth of 5m from the Western Channel Observatory (WCO; http://www.westernchannelobservatory. org.uk/) coastal station ‘L4’ (50°15.00N; 4°13.00W) on the following dates: 2018-09-24, 2018-10-17, 2018-11-05, 2019-02-11, 2019-03-11, 2019-04-01, 2019-07-22 (for details see Supplementary Table 2). Seawater was transferred immediately to a clean 2 L acid-washed polycarbonate (PC) Nalgene bottles (Thermo Fisher Scientific, Waltham, USA) and placed in a cooler box (Igloo, Katy, USA) at ambient temperature. Upon return to shore, water was transported to the University of Exeter for immediate processing (Two hours maximum duration from collection to processing).

*Isolation of SAR11 strain H2P3a and OM43 strains*: 1 L seawater was collected as described above in September 2017, filtered through a 142 mm Merck Millipore pore-size 0.2 µm PC filter and autoclaved in 2 L Duran bottles (DWK Life Sciences GmbH, Mainz, Germany) with the lid tightly shut to maintain carbon chemistry [[1]](https://paperpile.com/c/Ax3mHx/NLqrv). Upon cooling, nutrients were added to make natural seawater medium (NSW): 1 mM NH_4_Cl, 10 µM K_2_HPO_4_, 1 µM FeCl_3_, 25 µM glycine, 25 µM methionine, 100 µM pyruvate [[2]](https://paperpile.com/c/Ax3mHx/mQKYH). 1 mL of medium was placed into each 2 mL well in three sterile 96-well acid washed Teflon plates (Radleys, UK). Surface water from station L4 was filtered through a 142 mm Merck Millipore 0.2 µm PC filter to remove larger plankton and bacteria and enrich for smaller bacteria. A 200 µL aliquot was stained with SybrGreen and then quantified on a C6 Accuri flow cytometer. The remaining retained fraction was diluted in NSW to a density of ~ 1 cell·µL^-1^. 1 µL of diluted inoculum was added to each well, with eight wells left blank as negative controls and eight wells with undiluted low-nucleic acid community fraction as positive controls. Plates were covered and incubated in the dark at 15 °C for three months and checked monthly for positive growth by flow cytometry as described above. Positive wells (>10^6^ cells·mL^-1^) were transferred into 50 mL cultures in NSW medium and passaged three times once they achieved >10^6^ cells·mL^-1^). To verify culture purity and to taxonomically classify new isolates, the 16S rRNA gene was amplified from 10 µL of exponentially growing culture, heated to 95°C for 10 minutes, followed by PCR (25 cycles, 30 s @ 98 °C; 45 s @ 59 °C; 45 s @ 72 °C; final extension of 90 s @ 72 °C) using commercially available 27F and 1492R primers by Eurofins Genomics. Amplicon DNA was purified for Sanger sequencing (Eurofins) using a QIAquick PCR purification kit (Qiagen) following the manufacturer’s standard protocol. 16S rRNA sequences were compared to known bacterial sequences in the SILVA SSU database with SINA [[3]](https://paperpile.com/c/Ax3mHx/Zdmww). Sequences were aligned using T-coffee [[4]](https://paperpile.com/c/Ax3mHx/ctsOU), curated using Gblocks [[5]](https://paperpile.com/c/Ax3mHx/XQqsK) and maximum likelihood trees for both alignments were created using PhyML with standard settings and 500 bootstraps [[6]](https://paperpile.com/c/Ax3mHx/TXdjP). The trees were visualised using FigTree (v1.4.4, available https://github.com/rambaut/figtree).

*Host cultivation:* SAR11 strains HTCC1062 and HTCC7211 were kindly provided by the Giovannoni lab (Oregon State University, USA) and were kept in continuous culture in 50 mL polycarbonate flasks at 15 °C on artificial seawater medium ASM1 [[7]](https://paperpile.com/c/Ax3mHx/vtgCR) throughout the duration of the study. Following isolation, cultures of OM43 and H2P3α were maintained on ASM1; medium for OM43 was further amended with 100 mM MeOH and 32 µL of commercially available MEM amino acid solution per 1.6 L of medium (Sigma, M5550), to improve cellular yields and reduce occurrences of chaining cells (observed through EM, Fig. 5, and a phenotype of alanine starvation in SAR11 [[2]](https://paperpile.com/c/Ax3mHx/mQKYH)), respectively.

*Viral concentration and inoculation:* Monthly samples of 2 L of surface water were collected from station L4 as described above and sequentially filtered through a 142 mm Whatman GF/D filter (2.7 µm pore size) and a 142 mm 0.2 µm pore polycarbonate filter (Merck Millipore) using a peristaltic pump, to remove larger sized bacterioplankton. The filtrate was concentrated to 50 mL (40-fold concentration) using a 50R VivaFlow tangential flow filtration unit (Sartorius Lab instruments, Goettingen, Germany). The concentrate was filtered through a 0.1 µm pore PVDF membrane syringe-filter to remove any residual small cells and used as inoculum for viral isolation. 1 mL of exponentially growing bacterial host in ASM1 medium (amended with nutrients for OM43 strains as described for host cultivation above) was placed into each 2 mL well of a 96-well, acid washed Teflon plate. Eight wells were used as blank medium controls (no cell controls). 100 µL of viral inoculum was added to each well in the 96-well plate, with eight wells amended with 10% (v/v) medium instead of viral inoculum to serve as no-virus controls. The plates were incubated for ~2 weeks until no-virus controls reached maximum cell density (~2 weeks for strains HTCC1062, HTCCC7211 and H2P3α; about 1 week for OM43 strains H5P1, C6P1, D12P1). Growth was monitored at the end of the incubation period by flow cytometry; incubation periods were determined by the average growth times and data of the strains used for infections. Cytograms of virus-amended wells and no-virus controls were compared to identify infections as described below. A detailed protocol of the viral isolation process is available on [www.protocols.io](http://www.protocols.io) (DOI: [dx.doi.org/10.17504/protocols.io.c36yrd](https://dx.doi.org/10.17504/protocols.io.c36yrd)). Once the no-virus controls reached maximum cell density, inoculated wells that did not show signs of infection were pooled (according to host type), filtered through a 0.1 µm pore PVDF membrane syringe (Durapore) filter to remove the cells and then used as fresh inoculum in a new 96-well plate of target host. This was repeated up to three times to amplify host-specific viral particles to a density whereby signs of viral infectivity could be observed on the flow cytometer.

*Identification of positive infection via flow cytometry:* We observed positive infections in cells via flow cytometry by comparing cytograms of wells inoculated with viruses against no-virus controls. Cytograms of wells containing infected cultures were identifiable by (1) a population shift of up to a 10-fold increase in green fluorescence compared to uninfected cultures; (2) an increase in the ‘noise’ fraction (presumably from cellular debris following lysis as well as viral particles) over time (Supplementary Video 1); and (3) a reduced maximum cell density (ranging from 10 to 1000-fold depending on initial cell density and viral inoculum concentration) in infected cultures compared to no-virus controls (Fig. 1B). The presence of viral-like particles in wells where a shift in green fluorescence, increased noise, and reduced maximum cell density were observed, were verified by TEM and reinfection of new cultures in fresh medium.

*Viral purification:* Following identification of a positive infection within a well, the contents of the well were filtered through a 0.1 µm pore-size PVDF syringe filter (Durapore) and used as inoculum in three rounds of viral purification. Briefly, a 96-well Teflon plate was inoculated with bacterial hosts as described previously. A 10-fold serial dilution series (from 10^0^-10^-10^) of the viral inoculum in ASM1 was added to a single row of the plate at 10 % v/v, with one well per row as no-virus controls (total of eight no-virus controls/plate). Plates were incubated at 18 °C for ~2 weeks until the no-virus controls reached maximum cell density and then screened for signs of viral infection using flow cytometry as described above. For each dilution series, the well amended with the lowest number of viruses that showed positive signs of infection was identified, and used as the inoculum in another round of viral purification [[8]](https://paperpile.com/c/Ax3mHx/F0XHY). Using this format, we were able to purify eight to twelve viral isolates simultaneously per plate.

*Viral isolation costs and handling time*. For each sample (processed environmental water sample), all steps of the initial viral isolation process (counting one to two plates for a total of three times) required ~6 hours run time on a flow cytometer. For the subsequent three rounds of purification, one plate was required for each host-sample combination (another ~6 hours of cytometer run time in total for one host-sample combination). Initial isolation of viruses from environmental water samples and three rounds of purification took ~10 weeks of incubation time in total. Between all steps, our protocol required ~7 hours of handling time per sample. Following three rounds of viral purification, generating sufficiently high viral titres to extract enough DNA for sequencing (approximately two weeks incubation time, and roughly four hours handling time over two days) was the rate-limiting step, and so required subselection of available viruses for sequencing. Future advances in DNA extraction efficiency, reducing DNA input requirements for sequencing and/or automation of viral DNA extraction will enable all isolated viruses to be sequenced. We estimate the cost of isolating a single virus is ~£20 for cultivation, flow cytometry and DNA extraction consumables, and ~£50-100 for genome sequencing at 30-fold coverage required for successful assembly, giving a total cost of £70-120 per sequenced viral isolate, not including the costs for person-hours. Thus, our protocol provides a high-throughput and scalable approach to viral isolation.

*Transmission Electron Microscopy of viral isolates.* For ultrastructural analysis, virus particles were transferred onto pioloform-coated electron microscopy (EM) copper grids (Agar Scientific, Standsted, UK) by floating the grids on droplets of virus-containing suspension for 3 min. Following a series of four washes on droplets of deionized water, the bound virus particles were contrasted with 2 % (w/v) uranyl acetate in 2 % (w/v) methyl cellulose (mixed 1:9) on ice for 8 min and the grids then air-dried on a wire loop after carefully removing excess staining solution with a filter. Dried grids were inspected with a JEOL JEM 1400 transmission electron microscope operated at 120 kV and images taken with a digital camera (ES 1000W CCD, Gatan, Abingdon, UK).

*Host ranges of viral isolates*. To test the infectivity of phage isolates against different hosts, 2 mL 96-well Teflon plates were prepared with 1 mL of exponentially growing bacterial host (HTCC1062, HTCC7211, H2P3α) in ASM1 medium. As medium control, ASM1 without any bacteria was added to eight wells. 100 µL of phage inoculum was added to eight wells per host type. As no-virus control, for each host further eight wells were left without any viruses added to them. The plate was incubated for ~2 weeks and analysed for signs of infection using flow cytometry as described above. A phage was classified as unable to infect a certain host if none of the eight amended replicates showed signs of infection.

*DNA preparation and sequencing of viral isolates.* 50 mL ASM1 (amended with 100 mM MeOH and amino acid solution for OM43 as described above) in 250 mL acid-washed, polycarbonate flasks were inoculated with host cultures to 10^6^ cells·mL^-1^. The cultures were infected with 10% v/v viruses in ASM1 medium and incubated until after host cell lysis was detected using flow cytometry. The cultures were transferred to 50 mL Falcon tubes and the cellular fraction was removed by centrifugation (GSA rotor, Thermo Scientific 75007588) at 8,500 rpm/10,015 x g for 120 minutes. The supernatant was subsequently filtered through pore-size 0.1 µm PVDF syringe filter membranes to remove any remaining smaller cellular debris. Phages in the filtrate were precipitated using a modified version of an established PEG8000/NaCl DNA isolation method [[9]](https://paperpile.com/c/Ax3mHx/oiatB). Briefly, the filtered phage lysate was transferred into 50 mL Falcon tubes with pre-weighted 5 g PEG8000 and 3.3 g NaCl, shaken until both dissolved and incubated on ice overnight. The phage particles were then pelleted by centrifugation at 8,500 rpm/10,015 x g for 90 min at 4 °C. Supernatant was discarded and phage particles resuspended by rinsing Falcon tubes twice with 1 mL SM buffer (100 mM NaCl, 8 mM MgSO_4_·7H_2_O, 50 mM Tris-Cl). DNA was extracted using the Wizard DNA Clean-Up system (Promega) following manufacturer’s instructions, but instead of TE we used pre-heated 60 °C nuclease free water to elute DNA. DNA was sequenced by MicrobesNG (Birmingham, UK) with Illumina paired-end HiSeq 2500. Default settings were used for all programs listed below, unless specified otherwise. All reads were trimmed, quality-controlled and error-corrected using bbmap and tadpole [[10]](https://paperpile.com/c/Ax3mHx/hkeu3). Contigs were assembled using SPAdes v3.13 [[11]](https://paperpile.com/c/Ax3mHx/NoFEh) and evaluated with QUAST [[12]](https://paperpile.com/c/Ax3mHx/T3VXI). The trimmed reads were mapped back against the contigs for scaffolding using bowtie2 [[13]](https://paperpile.com/c/Ax3mHx/k2gFp), BamM (alignment 0.9, identity 0.95) (available at <https://github.com/minillinim/BamM>) and samtools [[14]](https://paperpile.com/c/Ax3mHx/zTSf8). Viral contigs were confirmed with VirSorter (categories 1 or 2, >15kbp) and only accepted if the representative contig showed mean depth coverage an order of magnitude greater than the next highest recruiting contig (to filter out any cellular DNA carryover). Gene calls returned by VirSorter were imported into DNA Master for manual curation [[15]](https://paperpile.com/c/Ax3mHx/sYNqZ). Additional gene calls were made using GenMark [[16]](https://paperpile.com/c/Ax3mHx/t7UvQ), GenMarkS [[17]](https://paperpile.com/c/Ax3mHx/eJdIG), GenMarkS2 [[18]](https://paperpile.com/c/Ax3mHx/P92yr), GenMark.hmm [[19]](https://paperpile.com/c/Ax3mHx/R20Db), GenMark.heuristic [[20]](https://paperpile.com/c/Ax3mHx/aQaAt), Glimmer v.3.02 [[21]](https://paperpile.com/c/Ax3mHx/grzVs), and Prodigal v.2.6.3 [[22]](https://paperpile.com/c/Ax3mHx/WHNt). All gene calls were listed and compared using a scoring system which evaluates gene length, gene overlap and coding potential of ORFs [[15]](https://paperpile.com/c/Ax3mHx/sYNqZ). ORFs were then annotated using BLASTp against the NCBI’s non-redundant protein sequences [[23]](https://paperpile.com/c/Ax3mHx/g98nd), Phmmer [[24]](https://paperpile.com/c/Ax3mHx/3ghlj) against UniProts UniProtKB and uniprotrefprot [[25]](https://paperpile.com/c/Ax3mHx/udXdK), Swissprot [[26]](https://paperpile.com/c/Ax3mHx/Eud05) as well as InterProScan [[27]](https://paperpile.com/c/Ax3mHx/P5H9e) and Pfam [[28]](https://paperpile.com/c/Ax3mHx/n3xgj). Virally-encoded tRNAs were identified with tRNAScan-SE v2.0 using settings `tRNAscan-SE -qQ --detail -o# -m# -f# -l# -c [tRNAscan-SE.conf](http://lowelab.ucsc.edu/download/tRNAscan-SE.conf) -s# -B` [[29]](https://paperpile.com/c/Ax3mHx/juoWD) to identify putative tRNA genes. Matching tRNA genes (e.g. viral and host tRNA-Leu) were then aligned using BLASTn [[30]](https://paperpile.com/c/Ax3mHx/ATFv). All viral genomes were organised into viral clusters (ICTV-recognised genera) based on shared gene content with VConTACT2 [[31]](https://paperpile.com/c/Ax3mHx/6qrVI), and into populations using a boundary cutoff of 95% ANI over 85% contig length [[32]](https://paperpile.com/c/Ax3mHx/Gl33h).

*Evaluation of gene-sharing networks with hypergeometric testing*. For the hypergeometric testing we gathered all annotated genomes from this study, other known pelagiphage isolates [[33, 34]](https://paperpile.com/c/Ax3mHx/3Rhj+tc4G) and we arbitrarily picked one representative contig from each cluster of complete phage genomes that were retrieved from a metagenomic Mediterranean deep chlorophyll maximum sample, that the authors speculated to be infective towards 1A SAR11s and included them in the phylogenetic analysis [[35]](https://paperpile.com/c/Ax3mHx/V4dd). The contigs were imported into anvio v6.1 [[36]](https://paperpile.com/c/Ax3mHx/biybr) where open reading frames were identified internally using Prodigal v2.6.3. The contigs were then used to run a pangenome analysis using the --use-ncbi-blast option. The binning function was then used to export the protein clusters, from which a matrix of the sum of shared proteins between all phages was created. The hypergeom function in the sciPy.stats python package was used to calculate the probability of sharing a protein between phages, based on which we created an average correlation cluster map.

*Viral phylogenetic analysis.* First we identified additional contigs to include in the phylogenetic analysis. The Global Ocean Virome (GOV2) [[37]](https://paperpile.com/c/Ax3mHx/Jq1ZK) and a virome from the Western English Channel [[38]](https://paperpile.com/c/Ax3mHx/ilp3Z) were screened for contigs that belonged to the same population (95% ANI over 80% length using ClusterGenomes.pl - https://github.com/simroux/ClusterGenomes) or the same genus (clustered by VConTACT2 [[39]](https://paperpile.com/c/Ax3mHx/HPgGG) into the same viral cluster, using default parameters) as isolate genomes. Additionally, all available genomes from Pelagibacter phage isolates were included, as well as selected fosmid derived contigs from Mediterranean metagenomes and 26 sequences from metagenomic mining that were identified as putative Pelagimyophages [[33–35, 40, 41]](https://paperpile.com/c/Ax3mHx/V4dd+3Rhj+tc4G+JTj6+x39l). All sequences were subjected to gene calling using Prodigal v2.6.3. (default parameters) and screened for TerL genes by aligning all proteins against known TerL genes in annotated genomes (belonging to clusters A, B and D, as well as siphovirus *Kolga*) from this study using Protein BLAST (default parameter) [[22, 30]](https://paperpile.com/c/Ax3mHx/WHNt+ATFv). The TerL gene of Bordetella phage LK3 was added as an outgroup. The TerL genes were aligned within the Phylogeny.fr online service [[42]](https://paperpile.com/c/Ax3mHx/E96lq) opting for MUSCLE alignment [[43]](https://paperpile.com/c/Ax3mHx/Ra0D) and Gblocks curation [[5]](https://paperpile.com/c/Ax3mHx/XQqsK) with default settings. A maximum-likelihood tree was calculated with PhyML [[44, 45]](https://paperpile.com/c/Ax3mHx/EHYX+xmGV), visualised using FigTree (v.1.4.4, available at <http://tree.bio.ed.ac.uk/software/figtree/>) and edited in Inkscape ([www.inkscape.org](http://www.inkscape.org)) for aesthetics (font adjustments and addition of GOV2 metadata).

We only identified a single additional contig from metagenomes using the approach described above that was similar to OM43 phage *Venkman*. Only one other known phage for a related host (LD28 phage P19250A [[46]](https://paperpile.com/c/Ax3mHx/8ed7A)) was available. These three contigs as well as all 14,883 viral contigs publicly available in January 2020 on<http://millardlab.org/> were gene called using Prodigal v2.6.3. [[22]](https://paperpile.com/c/Ax3mHx/WHNt). All called genes were compared to vFams [[47]](https://paperpile.com/c/Ax3mHx/PikiM) using hmmsearch [[48]](https://paperpile.com/c/Ax3mHx/mR4hZ) and sorted by E-value for best hits. The best matching gene sequences that were identified as similar to shared vFams found in *Venkman* and P19250A were important to the Phylogeny.fr online service [[42]](https://paperpile.com/c/Ax3mHx/E96lq). Sequences of were aligned individually using T-coffee [[4]](https://paperpile.com/c/Ax3mHx/ctsOU), curated using Gblocks [[5]](https://paperpile.com/c/Ax3mHx/XQqsK) and concatenated manually. Maximum likelihood trees for both alignments were created using PhyML with standard settings and 500 bootstraps [[6, 44]](https://paperpile.com/c/Ax3mHx/TXdjP+EHYX). The trees were visualised with FigTree (v.1.4.4) and edited in Inkscape ([www.inkscape.org](http://www.inkscape.org)) for aesthetics. All sequences used for phylogenetic analysis are available at <https://github.com/ViralPirates/viral-dte>.

*Calculation of Average Nucleotide Identity*. All-vs-all average nucleotide identity (ANI) for our new isolates and existing pelagiphages using nucmer v.4.1 with the --nooptimize flag, followed by show-coords to convert the delta file into tabular format. ANI was calculated by taking the mean of all matching regions between two isolates.

*Viral metagenomic recruitment.* For assessing relative abundances of phage contigs in global datasets, a virome from the Western English Channel and all samples of the Global Ocean Virome dataset (GOV2) were used for recruitment [[37, 38]](https://paperpile.com/c/Ax3mHx/ilp3Z+Jq1ZK). Metagenomic reads were downloaded from the European Nucleotide Archive ERR2625613 and subsampled to 5 million reads using bbmap’s reformat.sh command. A bowtie2 [[13]](https://paperpile.com/c/Ax3mHx/k2gFp) index of dereplicated contigs was created from 25 known pelagiphage genomes from isolates [[33, 34, 41]](https://paperpile.com/c/Ax3mHx/3Rhj+tc4G+x39l), LD28 phage P19250A [[46]](https://paperpile.com/c/Ax3mHx/8ed7A) and from this study six viral population representative genomes isolated on SAR11 hosts and one Methylophilales phage *Venkman* isolated on OM43 host. Reads were mapped against the genomes for each metagenome sample using bowtie2 using these commands: bowtie2 --seed 42 --non-deterministic. To calculate coverage and Reads Per Kilobase of contig per Million reads (RPKM) we used coverm [reference], with the following commands: coverm contig --bam-files *.bam --min-read-percent-identity 0.9 --methods rpkm --min-covered-fraction 0.4. To minimize false positive detection rates [[32]](https://paperpile.com/c/Ax3mHx/Gl33h), contigs that did not meet the 40% minimum genome coverage in a given sample were given an RPKM value of 0.

*Evaluation of isolation efficiency as a function of matching host and virus geography.* In total, 105 attempts were made for isolating viruses for SAR11 across the three host strains (HTCC7211: 35, HTCC1062: 40; H2P3α: 30). For each strain, the number of successful attempts were recorded in a vector, *v_s_*, of length *n* where *n* is the number of attempts made to isolate a virus, such that *v_i_*=1 if a successful attempt was made and *v_i_*=0 if an unsuccessful attempt was made. To test whether observed rates of success for H2P3 were significantly elevated compared to those for HTCC1062 and HTCC7211, the observed rates were evaluated against the null hypothesis that host strain did not make a difference to isolation success. Briefly, for each pair (H2P3 vs HTCC1062; H2P3α vs HTCC7211), the two vectors *v_s1_ and v_s2_* were concatenated, and then randomly subsampled 999 times with replacement to a size of the smallest value of *n* between the vectors. The number of successes in each subsample were counted and the distribution of values across the 999 bootstraps was recorded. *P-*values were calculated by evaluating how many times within the 999 bootstraps a value as extreme as the observed value had been recorded.

*16S rRNA high-throughput amplicon sequencing and analysis*. Genomic DNA was extracted using Qiagen DNeasy PowerWater Kit (REF 14900-50-NF) from biomass retained on 0.2 µm PC filters following the manufacturer's protocol with minor modifications to increase the yield. Step 7 was modified from a 5 minute to 10 minute vortex bead beat. Step 21 was changed to have a 2 minute incubation with EB warmed to 55C. Amplification of the hypervariable V4 region of the 16S rRNA gene was performed using the 515fB (5'-GTGYCAGCMGCCGCGGTAA-3') and 806rB (5'-GGACTACNVGGGTWTCTAAT-3') primers. Libraries for each reaction were done attaching dual indices and Illumina adapters with the NexTera XT Index Kit (Illumina Inc.) Purified libraries were pooled and sequenced in a MiSeq platform. Primer sequences from paired-end fastq files were trimmed using CutAdapt [[49]](https://paperpile.com/c/Ax3mHx/uCYz8). Trimmed sequences were quality filtered, dereplicated and merged with DADA2 R package (version 1.8 [[50]](https://paperpile.com/c/Ax3mHx/fxs0M)). Taxonomic assignment was performed using the “assignTaxonomy” command within dada2 pipeline and silva_nr_v132 database [[51]](https://paperpile.com/c/Ax3mHx/k1JBe) as training set. SAR11 ASVs were extracted for further characterization using Phyloassigner v6.166 [[52]](https://paperpile.com/c/Ax3mHx/Ipmht) and oligotyping [[53]](https://paperpile.com/c/Ax3mHx/DTaOZ). SAR11 phylogenetic database SAR11_Phy_DB used in this study is available at [https://www.github.com/lbolanos32/NAAMES_2020](https://github.com/lbolanos32/NAAMES_2020). Relative contribution barplots were done in R using ggplot2 [[54]](https://paperpile.com/c/Ax3mHx/julF3) and edited in inkscape ([www.inkscape.org](http://www.inkscape.org)) for aesthetics.

**Supplementary References**

1. [Henson MW, Pitre DM, Weckhorst JL, Celeste Lanclos V, Webber AT, Cameron Thrash J. Artificial Seawater Media Facilitate Cultivating Members of the Microbial Majority from the Gulf of Mexico. *American Society for Microbiology* 2016; **1**: 1–9.](http://paperpile.com/b/Ax3mHx/NLqrv)

2. [Carini P, Steindler L, Beszteri S, Giovannoni SJ. Nutrient requirements for growth of the extreme oligotroph ‘Candidatus Pelagibacter ubique’ HTCC1062 on a defined medium. *ISME J* 2013; **7**: 592–602.](http://paperpile.com/b/Ax3mHx/mQKYH)

3. [Pruesse E, Peplies J, Glöckner FO. SINA: accurate high-throughput multiple sequence alignment of ribosomal RNA genes. *Bioinformatics* 2012; **28**: 1823–1829.](http://paperpile.com/b/Ax3mHx/Zdmww)

4. [Notredame C, Higgins DG, Heringa J. T-Coffee: A novel method for fast and accurate multiple sequence alignment. *J Mol Biol* 2000; **302**: 205–217.](http://paperpile.com/b/Ax3mHx/ctsOU)

5. [Talavera G, Castresana J. Improvement of phylogenies after removing divergent and ambiguously aligned blocks from protein sequence alignments. *Syst Biol* 2007; **56**: 564–577.](http://paperpile.com/b/Ax3mHx/XQqsK)

6. [Guindon S, Lethiec F, Duroux P, Gascuel O. PHYML Online--a web server for fast maximum likelihood-based phylogenetic inference. *Nucleic Acids Res* 2005; **33**: W557–9.](http://paperpile.com/b/Ax3mHx/TXdjP)

7. [Carini P, Campbell EO, Morré J, Sañudo-Wilhelmy SA, Thrash JC, Bennett SE, et al. Discovery of a SAR11 growth requirement for thiamin’s pyrimidine precursor and its distribution in the Sargasso Sea. *ISME J* 2014; **8**: 1727–1738.](http://paperpile.com/b/Ax3mHx/vtgCR)

8. [Nagasaki K, Bratbak G. Isolation of viruses infecting photosynthetic and nonphotosynthetic protists. *Manual of aquatic viral ecology ASLO* 2010; 92–101.](http://paperpile.com/b/Ax3mHx/F0XHY)

9. [Solonenko N. Isolation of DNA from phage lysate. *protocols.io* 2016.](http://paperpile.com/b/Ax3mHx/oiatB)

10. [Bushnell B, Rood J, Singer E. BBMerge – Accurate paired shotgun read merging via overlap. *PLOS ONE* . 2017. , **12**: e0185056](http://paperpile.com/b/Ax3mHx/hkeu3)

11. [Bankevich A, Nurk S, Antipov D, Gurevich AA, Dvorkin M, Kulikov AS, et al. SPAdes: a new genome assembly algorithm and its applications to single-cell sequencing. *J Comput Biol* 2012; **19**: 455–477.](http://paperpile.com/b/Ax3mHx/NoFEh)

12. [Gurevich A, Saveliev V, Vyahhi N, Tesler G. QUAST: quality assessment tool for genome assemblies. *Bioinformatics* 2013; **29**: 1072–1075.](http://paperpile.com/b/Ax3mHx/T3VXI)

13. [Langmead B, Salzberg SL. Fast gapped-read alignment with Bowtie 2. *Nat Methods* 2012; **9**: 357–359.](http://paperpile.com/b/Ax3mHx/k2gFp)

14. [Li H, Handsaker B, Wysoker A, Fennell T, Ruan J, Homer N, et al. The Sequence Alignment/Map format and SAMtools. *Bioinformatics* 2009; **25**: 2078–2079.](http://paperpile.com/b/Ax3mHx/zTSf8)

15. [Salisbury A, Tsourkas PK. A Method for Improving the Accuracy and Efficiency of Bacteriophage Genome Annotation. *Int J Mol Sci* 2019; **20**.](http://paperpile.com/b/Ax3mHx/sYNqZ)

16. [Borodovsky M, McIninch J. GenMark: Parallel gene recognition for both DNA strands. *Computers Chemistry* 1993; **17**: 123–133.](http://paperpile.com/b/Ax3mHx/t7UvQ)

17. [Besemer J, Lomsadze A, Borodovsky M. GeneMarkS: a self-training method for prediction of gene starts in microbial genomes. Implications for finding sequence motifs in regulatory regions. *Nucleic Acids Res* 2001; **29**: 2607–2618.](http://paperpile.com/b/Ax3mHx/eJdIG)

18. [Lomsadze A, Gemayel K, Tang S, Borodovsky M. Modeling leaderless transcription and atypical genes results in more accurate gene prediction in prokaryotes. *Genome Res* 2018; **28**: 1079–1089.](http://paperpile.com/b/Ax3mHx/P92yr)

19. [Zhu W, Lomsadze A, Borodovsky M. Ab initio gene identification in metagenomic sequences. *Nucleic Acids Res* 2010; **38**: e132.](http://paperpile.com/b/Ax3mHx/R20Db)

20. [Besemer J, Borodovsky M. Heuristic approach to deriving models for gene finding. *Nucleic Acids Res* 1999; **27**: 3911–3920.](http://paperpile.com/b/Ax3mHx/aQaAt)

21. [Delcher AL, Bratke KA, Powers EC, Salzberg SL. Identifying bacterial genes and endosymbiont DNA with Glimmer. *Bioinformatics* 2007; **23**: 673–679.](http://paperpile.com/b/Ax3mHx/grzVs)

22. [Hyatt D, Chen G-L, Locascio PF, Land ML, Larimer FW, Hauser LJ. Prodigal: prokaryotic gene recognition and translation initiation site identification. *BMC Bioinformatics* 2010; **11**: 119.](http://paperpile.com/b/Ax3mHx/WHNt)

23. [Pruitt KD, Tatusova T, Maglott DR. NCBI reference sequences (RefSeq): a curated non-redundant sequence database of genomes, transcripts and proteins. *Nucleic Acids Res* 2007; **35**: D61–5.](http://paperpile.com/b/Ax3mHx/g98nd)

24. [Potter SC, Luciani A, Eddy SR, Park Y, Lopez R, Finn RD. HMMER web server: 2018 update. *Nucleic Acids Res* 2018; **46**: W200–W204.](http://paperpile.com/b/Ax3mHx/3ghlj)

25. [UniProt Consortium. UniProt: a worldwide hub of protein knowledge. *Nucleic Acids Res* 2019; **47**: D506–D515.](http://paperpile.com/b/Ax3mHx/udXdK)

26. [Bairoch A, Apweiler R. The SWISS-PROT protein sequence database and its supplement TrEMBL in 2000. *Nucleic Acids Res* 2000; **28**: 45–48.](http://paperpile.com/b/Ax3mHx/Eud05)

27. [Jones P, Binns D, Chang H-Y, Fraser M, Li W, McAnulla C, et al. InterProScan 5: genome-scale protein function classification. *Bioinformatics* 2014; **30**: 1236–1240.](http://paperpile.com/b/Ax3mHx/P5H9e)

28. [Finn RD, Bateman A, Clements J, Coggill P, Eberhardt RY, Eddy SR, et al. Pfam: the protein families database. *Nucleic Acids Res* 2014; **42**: D222–30.](http://paperpile.com/b/Ax3mHx/n3xgj)

29. [Lowe TM, Chan PP. tRNAscan-SE On-line: integrating search and context for analysis of transfer RNA genes. *Nucleic Acids Res* 2016; **44**: W54–7.](http://paperpile.com/b/Ax3mHx/juoWD)

30. [Altschul SF, Gish W, Miller W, Myers EW, Lipman DJ. Basic local alignment search tool. *J Mol Biol* 1990; **215**: 403–410.](http://paperpile.com/b/Ax3mHx/ATFv)

31. [Bin Jang H, Bolduc B, Zablocki O, Kuhn JH, Roux S, Adriaenssens EM, et al. Taxonomic assignment of uncultivated prokaryotic virus genomes is enabled by gene-sharing networks. *Nat Biotechnol* 2019; **37**: 632–639.](http://paperpile.com/b/Ax3mHx/6qrVI)

32. [Roux S, Emerson JB, Eloe-Fadrosh EA, Sullivan MB. Benchmarking viromics: an in silico evaluation of metagenome-enabled estimates of viral community composition and diversity. *PeerJ* 2017; **5**: e3817.](http://paperpile.com/b/Ax3mHx/Gl33h)

33. [Zhao Y, Temperton B, Thrash JC, Schwalbach MS, Vergin KL, Landry ZC, et al. Abundant SAR11 viruses in the ocean. *Nature* 2013; **494**: 357–360.](http://paperpile.com/b/Ax3mHx/3Rhj)

34. [Zhao Y, Qin F, Zhang R, Giovannoni SJ, Zhang Z, Sun J, et al. Pelagiphages in the Podoviridae family integrate into host genomes. *Environ Microbiol* 2018.](http://paperpile.com/b/Ax3mHx/tc4G)

35. [Mizuno CM, Rodriguez-Valera F, Kimes NE, Ghai R. Expanding the marine virosphere using metagenomics. *PLoS Genet* 2013; **9**: e1003987.](http://paperpile.com/b/Ax3mHx/V4dd)

36. [Eren AM, Esen ÖC, Quince C, Vineis JH, Morrison HG, Sogin ML, et al. Anvi’o: an advanced analysis and visualization platform for 'omics data. *PeerJ* 2015; **3**: e1319.](http://paperpile.com/b/Ax3mHx/biybr)

37. [Gregory AC, Zayed AA, Sunagawa S, Wincker P, Sullivan MB, Temperton B, et al. Marine DNA Viral Macro- and Microdiversity from Pole to Pole. *Cell* 2019; **177**: 1109–1123.](http://paperpile.com/b/Ax3mHx/Jq1ZK)

38. [Warwick-Dugdale J, Solonenko N, Moore K, Chittick L, Gregory AC, Allen MJ, et al. Long-read metagenomics reveals cryptic and abundant marine viruses. *bioRxiv* . 2018. , 345041](http://paperpile.com/b/Ax3mHx/ilp3Z)

39. [Bolduc B, Jang HB, Doulcier G, You Z-Q, Roux S, Sullivan MB. vConTACT: an iVirus tool to classify double-stranded DNA viruses that infect Archaea and Bacteria. *PeerJ* 2017; **5**: e3243.](http://paperpile.com/b/Ax3mHx/HPgGG)

40. [Zaragoza-Solas A, Rodriguez-Valera F, López-Pérez M. Metagenome Mining Reveals Hidden Genomic Diversity of Pelagimyophages in Aquatic Environments. *mSystems* 2020; **5**.](http://paperpile.com/b/Ax3mHx/JTj6)

41. [Zhang Z, Qin F, Chen F, Chu X, Luo H, Zhang R, et al. Culturing novel and abundant pelagiphages in the ocean. *Environ Microbiol* 2020.](http://paperpile.com/b/Ax3mHx/x39l)

42. [Dereeper A, Guignon V, Blanc G, Audic S, Buffet S, Chevenet F, et al. Phylogeny.fr: robust phylogenetic analysis for the non-specialist. *Nucleic Acids Res* 2008; **36**: W465–9.](http://paperpile.com/b/Ax3mHx/E96lq)

43. [Edgar RC. MUSCLE: multiple sequence alignment with high accuracy and high throughput. *Nucleic Acids Res* 2004; **32**: 1792–1797.](http://paperpile.com/b/Ax3mHx/Ra0D)

44. [Anisimova M, Gascuel O. Approximate likelihood-ratio test for branches: A fast, accurate, and powerful alternative. *Syst Biol* 2006; **55**: 539–552.](http://paperpile.com/b/Ax3mHx/EHYX)

45. [Guindon S, Gascuel O. A simple, fast, and accurate algorithm to estimate large phylogenies by maximum likelihood. *Syst Biol* 2003; **52**: 696–704.](http://paperpile.com/b/Ax3mHx/xmGV)

46. [Moon K, Kang I, Kim S, Kim S-J, Cho J-C. Genome characteristics and environmental distribution of the first phage that infects the LD28 clade, a freshwater methylotrophic bacterial group. *Environ Microbiol* 2017; **19**: 4714–4727.](http://paperpile.com/b/Ax3mHx/8ed7A)

47. [Skewes-Cox P, Sharpton TJ, Pollard KS, DeRisi JL. Profile hidden Markov models for the detection of viruses within metagenomic sequence data. *PLoS One* 2014; **9**: e105067.](http://paperpile.com/b/Ax3mHx/PikiM)

48. [Eddy S. HMMER: profile HMMs for protein sequence analysis. 1998.](http://paperpile.com/b/Ax3mHx/mR4hZ)

49. [Martin M. Cutadapt removes adapter sequences from high-throughput sequencing reads. *EMBnet.journal* 2011; **17**: 10–12.](http://paperpile.com/b/Ax3mHx/uCYz8)

50. [Callahan BJ, McMurdie PJ, Rosen MJ, Han AW, Johnson AJA, Holmes SP. DADA2: High-resolution sample inference from Illumina amplicon data. *Nat Methods* 2016; **13**: 581–583.](http://paperpile.com/b/Ax3mHx/fxs0M)

51. [Quast C, Pruesse E, Yilmaz P, Gerken J, Schweer T, Yarza P, et al. The SILVA ribosomal RNA gene database project: improved data processing and web-based tools. *Nucleic Acids Res* 2013; **41**: D590–6.](http://paperpile.com/b/Ax3mHx/k1JBe)

52. [Vergin KL, Beszteri B, Monier A, Thrash JC, Temperton B, Treusch AH, et al. High-resolution SAR11 ecotype dynamics at the Bermuda Atlantic Time-series Study site by phylogenetic placement of pyrosequences. *ISME J* 2013; **7**: 1322–1332.](http://paperpile.com/b/Ax3mHx/Ipmht)

53. [Eren AM, Borisy GG, Huse SM, Mark Welch JL. Oligotyping analysis of the human oral microbiome. *Proc Natl Acad Sci U S A* 2014; **111**: E2875–84.](http://paperpile.com/b/Ax3mHx/DTaOZ)

54. [Wickham H. ggplot2: Elegant Graphics for Data Analysis. 2016. Springer-Verlag, New York.](http://paperpile.com/b/Ax3mHx/julF3)

**Supplementary Materials List**

Supplementary Fig. 1 Maximum likelihood trees (100,000 generations, sampled every 10 generations, first 5000 trees burn-in) of 16S rRNA genes of **(A)** Known SAR11 strains and new isolate H2P3α; **(B)** New OM43 isolates and other related members of Methylophilaceae. Trees in A and B were rooted using subclade III and the *Methylophilus*, respectively.

Supplementary Fig. 2 Cytograms of infected vs. uninfected SAR11 warm-water ecotype HTCC7211 cultures to isolate phages. Events within the acquisition range (“populations”) of no-virus controls range from 72.7% - 84.3% of total events, whereas infected cultures range from 27.7% - 78.6%. Note the increase in low fluorescence (FITC-A) events for phage cultures, which were used to identify infections in cultures.

Supplementary Fig. 3 Cytograms of infected vs. uninfected SAR11 cold-water ecotype HTCC1062 cultures to isolate phages. Events within the acquisition range (“populations”) of no-virus controls range from 72.7% - 80.5% of total events, whereas infected cultures range from 40.7% - 81.8%. Note the increase in low fluorescence (FITC-A) events for phage cultures, which were used to identify infections in cultures.

Supplementary Fig. 4 Cytograms of infected vs. uninfected new SAR11 isolate H2P3α cultures to isolate phages. Events within the acquisition range (“populations”) of no-virus controls range from 72.8% - 76.1% of total events, whereas infected cultures range from 16.5% - 72.0%. Note the increase in low fluorescence (FITC-A) events for phage cultures, which were used to identify infections in cultures.

Supplementary Fig. 5 Cytograms of infected vs. uninfected new OM43 isolate H5P1 cultures to isolate phages. Events within the acquisition range (“populations”) of no-virus controls range from 46.0% - 66.5% of total events, whereas infected cultures range from 15.7% - 56.8%. Note the increase in low fluorescence (FITC-A) events for phage cultures, which were used to identify infections in cultures. There was no clear discrimination between noise and the population peaks of the cytograms for infected cultures in March and April, therefore calculated percentages are likely influenced by application of gating parameters defined in other samples.

Supplementary Fig. 6 Distribution of pairwise average nucleotide identity (ANI) of all phages included in this study. The secondary peak at ANI >95% supports current use of this value as a cut-off for defining viral populations.

Supplementary Fig. 7 Unrooted maximum-likelihood tree of TerL genes found in *Pelagibacter* phages, using *Bordetella* phage LK3 as outgroup. Distinct branches were highlighted and labelled according to their respective hypergeometric cluster and viral family. Sampling environment of metagenomes used to identify pelagiphage-like contigs are marked: Epipelagic (TT-EPI mesopelagic (TT-MES), bathypelagic (BATHY), Southern Ocean/ Antarctica (ANT), Arctic Ocean (ARC). Branch support values are available within the newick file at <https://github.com/ViralPirates/viral-dte>.

Supplementary Fig. 8 Shared protein cluster network content of known phage isolates [[33, 34]](https://paperpile.com/c/Ax3mHx/3Rhj+tc4G) and our viral isolates against the NCBI Bacterial and Archaeal Viral RefSeq v85 with ICTV + NCBI taxonomy database (VConTACT2, default settings); different colours represent the ICTV-recognised genera assignments.

Supplementary Fig. 9 Relative abundance of SAR11 clade in Western English Channel at the time when samples were collected for viral isolation. (A) Total relative contribution. (B) Clade composition of the SAR11 fraction. The stacked barplots depict the relative contribution of each of the four clades to the total SAR11 fraction. NA: Not assigned to any of the four clades.

Supplementary Fig. 10 Growth curves of *Pelagibacter* *sp.* H2P3α, based on eight replicates in 125 mL flask of ASM1 medium, grown at different temperatures. Error bars represent ± std. error.

Supplementary Fig. 11 **(A)** Fluorescence and **(B)** Temperature at Western English Channel sampling station L4 over the course of the study. Grey dashed lines represent samples for viral isolation. Sampling missed phytoplankton blooms, potentially explaining the lower success of isolating viruses for OM43 strains.

Supplementary Fig. 12 Gene map of pelagiphages in Clusters A and B arranged inner to outer: *Eistla* (EXEVC025P), *Ran* (EXEVC014P), *Bylgja* (EXEVC010P) and *Eyrgjafa* (EXEVC018P). Solid lines indicate orthologous genes, dashed lines indicate genes of similar function.

Supplementary Fig. 13 Final cell densities of infected (blue) vs. uninfected (orange) hosts for 115 of the 117 viruses isolated in this study (two viruses isolated on OM43 D12P1 in July omitted). Dashed line at 10^6^ cells·mL^-1^ represents host density at T_0_. Points below this line indicate viruses reduced host density below the inoculum density. Named points refer to genomes sequenced in this study.

Supplementary Table 1 Pairwise average nucleotide identity of the 16S rRNA genes of OM43 isolates from this study.

Supplementary Table 2 Metadata associated with water samples taken for viral isolation.

Supplementary Table 3 Number of rounds of enrichment required for each water sample vs. target host combination before infection could be observed by flow cytometry.

Supplementary Table 4 Matrix of pairwise average nucleotide identity (ANI) for viruses from [[33, 34]](https://paperpile.com/c/Ax3mHx/3Rhj+tc4G) and this study.

Supplementary Table 5 General data and descriptions of sequenced viral isolates.

Supplementary Video 1 Time series of an infection of *Pelagibacter ubique* HTCC1062 showing how host cell density achieves a steady state in infected cultures that is lower than that of uninfected cultures. Associated cytograms are shown to demonstrate shifts in fluorescence associated with viral infection.
