# Supplementary figures and images for: Efficient dilution-to-extinction isolation of novel virus–host model systems for fastidious heterotrophic bacteria
Source: ISME J. 2021 Jan 25;15(6):1585–98. doi: 10.1038/s41396-020-00872-z (PMC8163748; doi:10.1038/s41396-020-00872-z)

**A**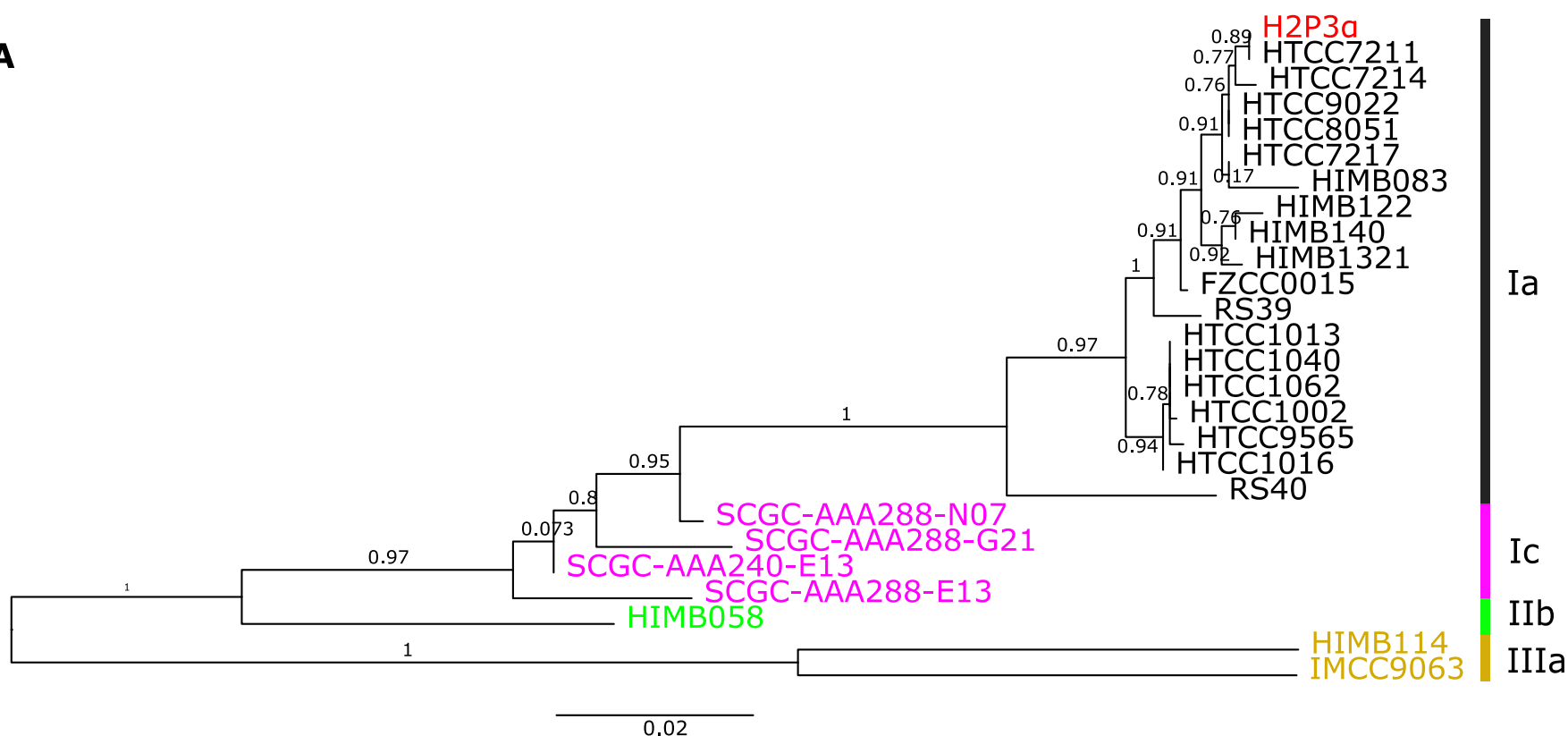**B**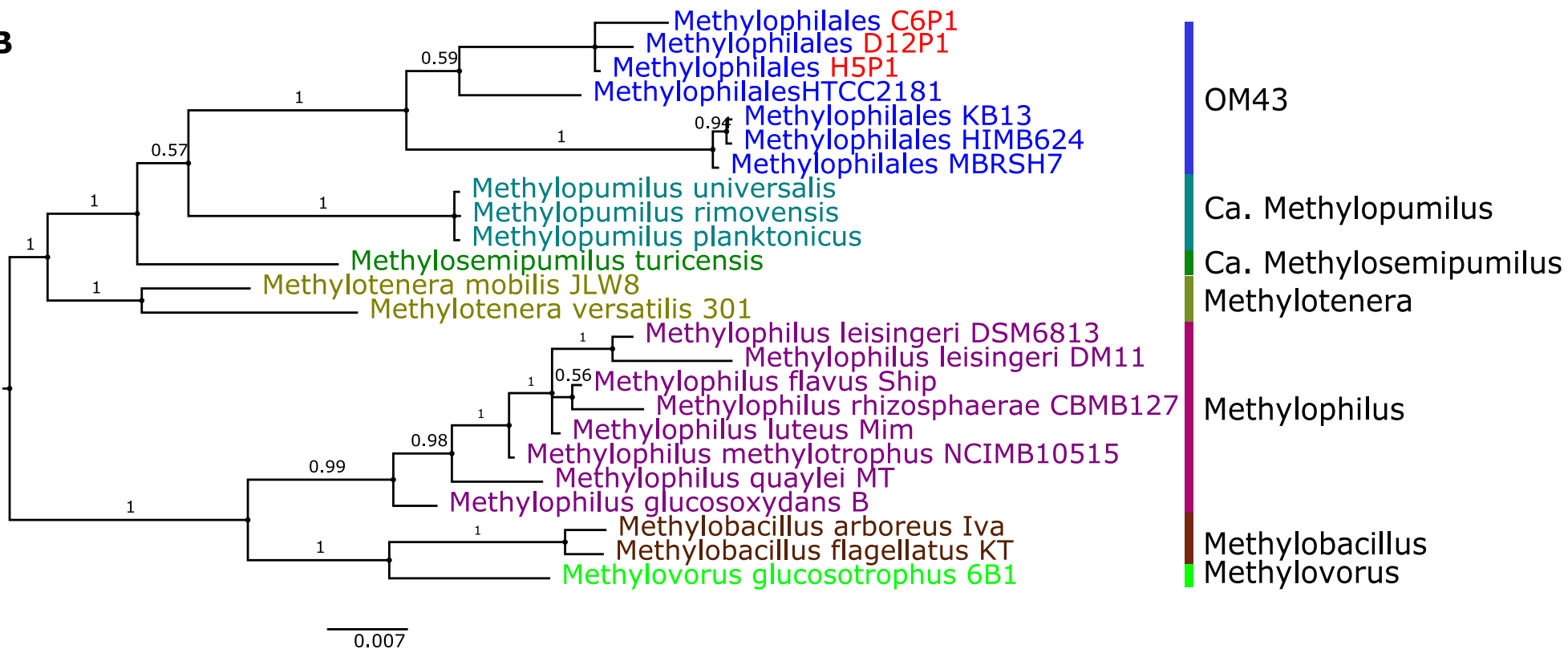

Supplement: Supplementary file 2 — Supplementary Figure 1 [file 41396_2020_872_MOESM2_ESM.pdf]

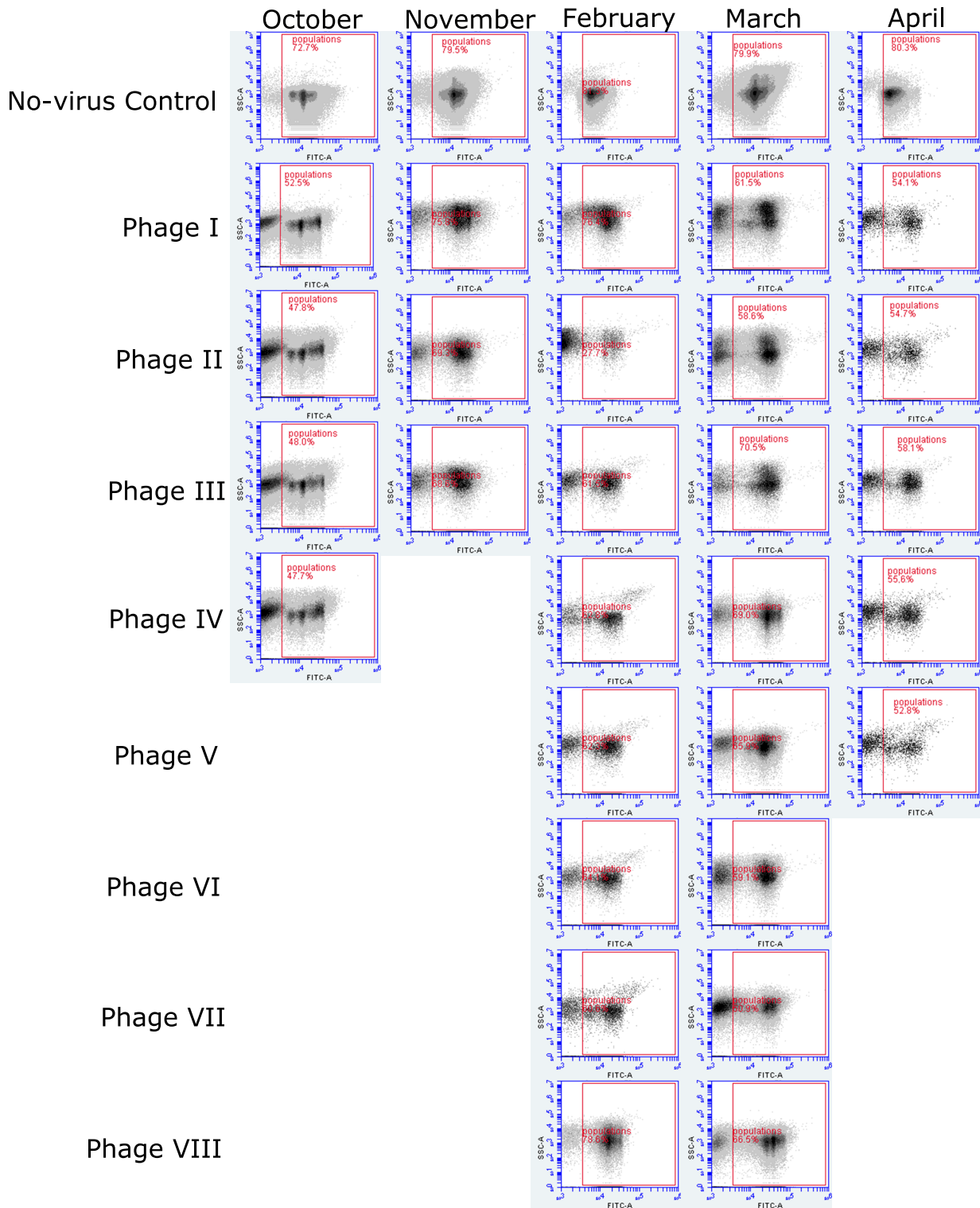

Supplement: Supplementary file 3 — Supplementary Figure 2 [file 41396_2020_872_MOESM3_ESM.pdf]

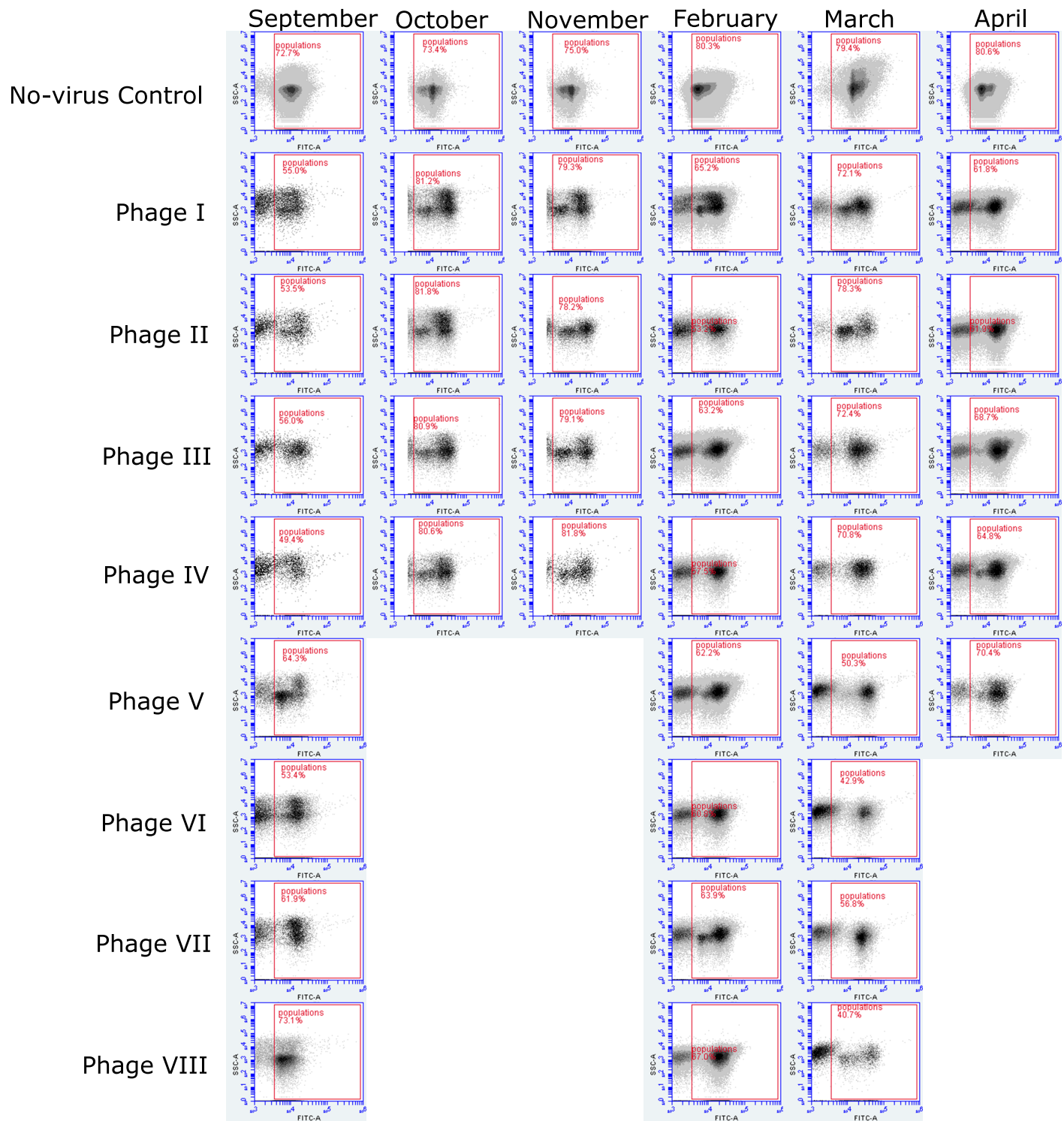

Supplement: Supplementary file 4 — Supplementary Figure 3 [file 41396_2020_872_MOESM4_ESM.pdf]

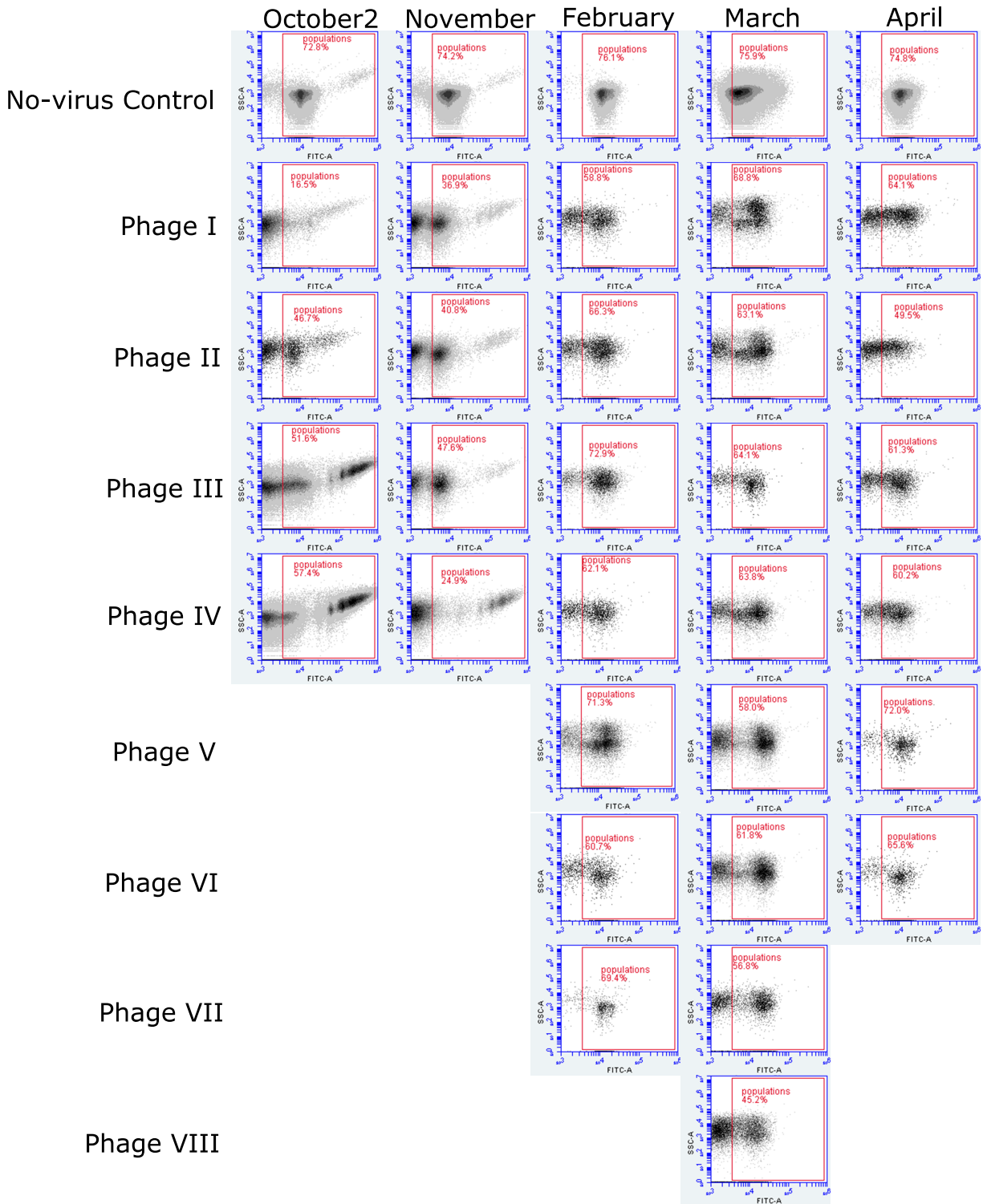

Supplement: Supplementary file 5 — Supplementary Figure 4 [file 41396_2020_872_MOESM5_ESM.pdf]

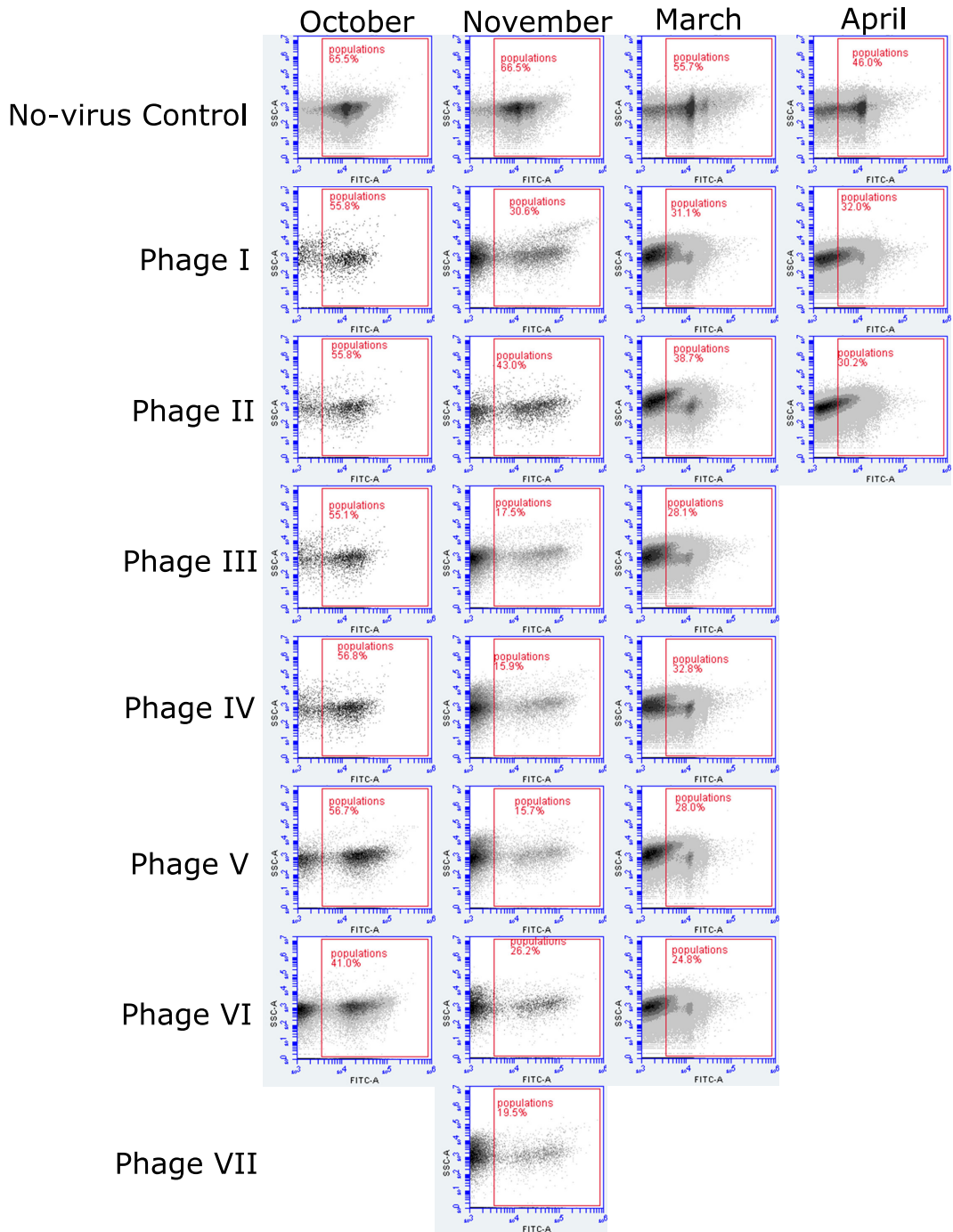

Supplement: Supplementary file 6 — Supplementary Figure 5 [file 41396_2020_872_MOESM6_ESM.pdf]

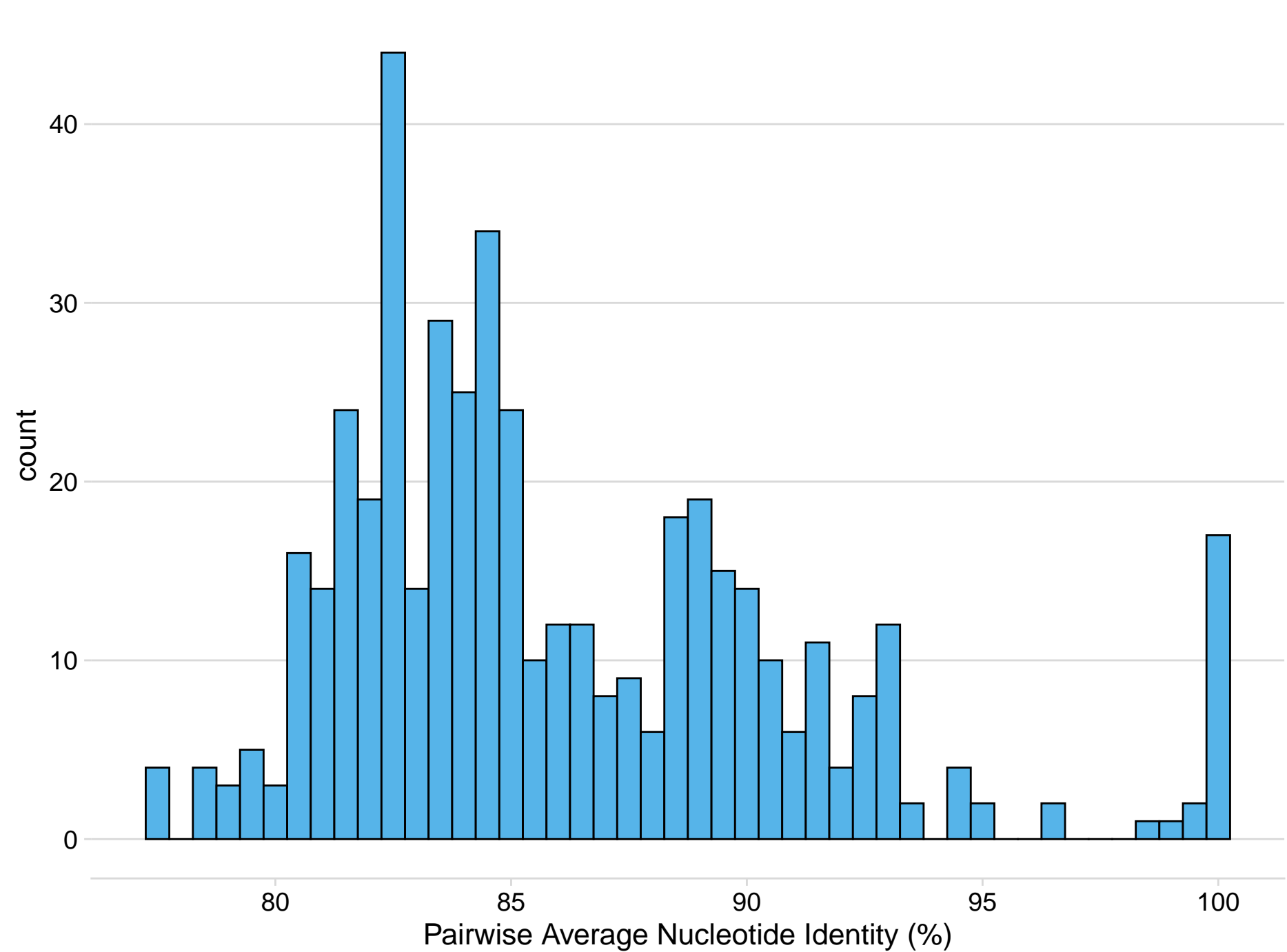

Supplement: Supplementary file 7 — Supplementary Figure 6 [file 41396_2020_872_MOESM7_ESM.pdf]

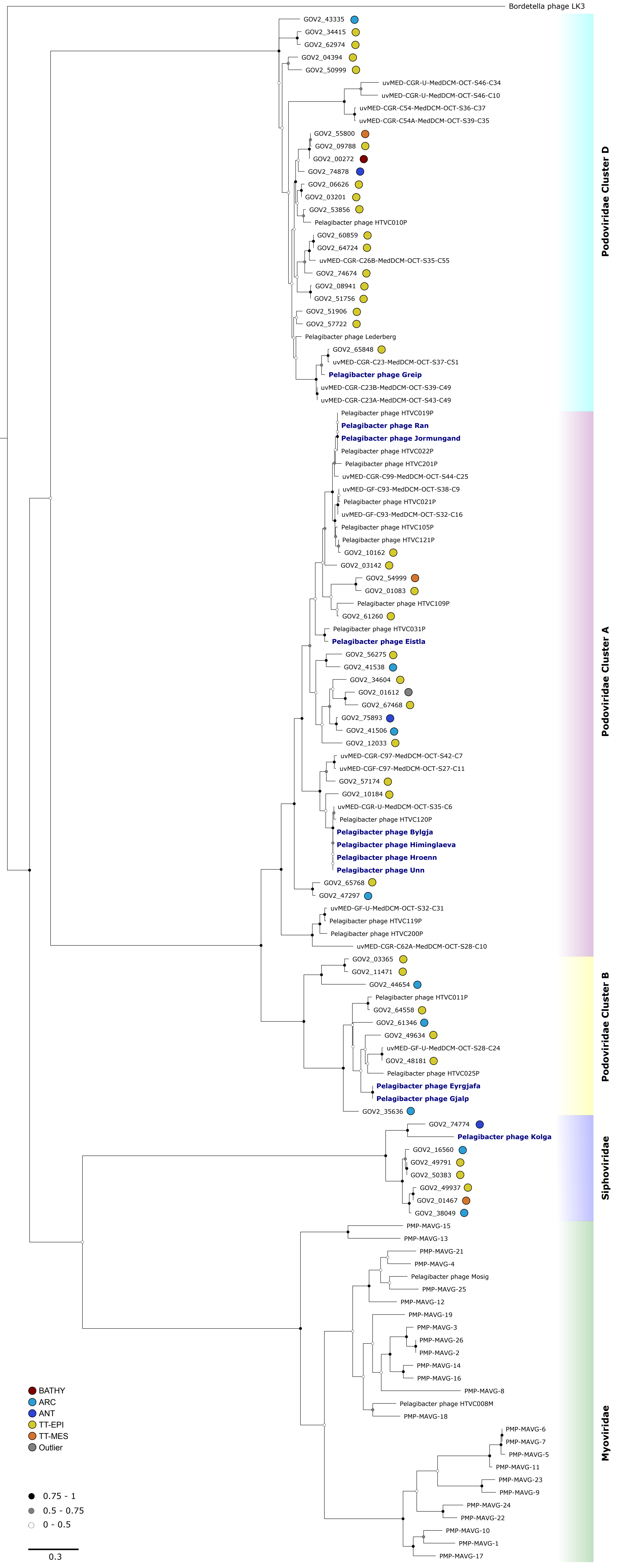

Supplement: Supplementary file 8 — Supplementary Figure 7 [file 41396_2020_872_MOESM8_ESM.pdf]

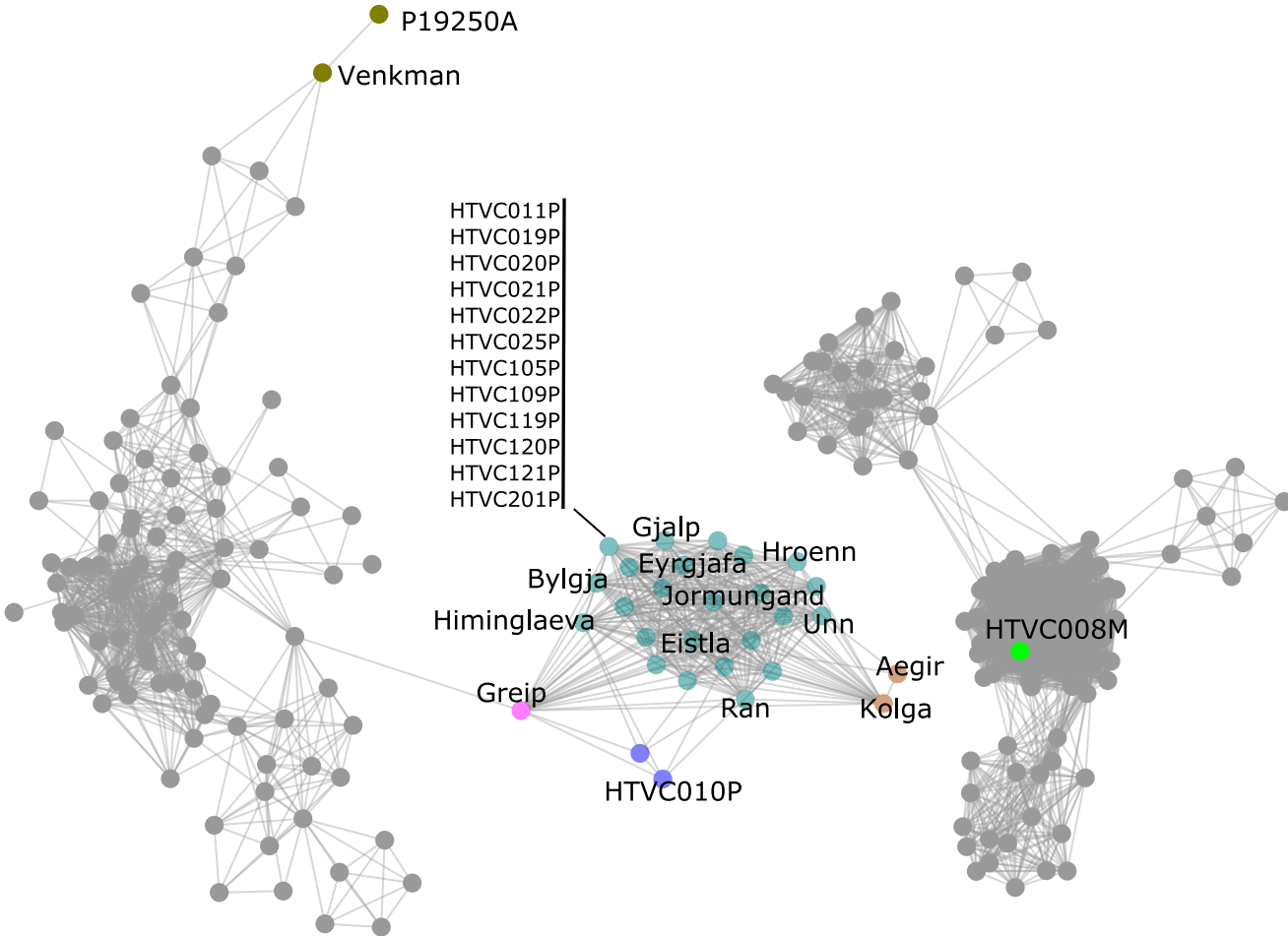

Supplement: Supplementary file 9 — Supplementary Figure 8 [file 41396_2020_872_MOESM9_ESM.pdf]

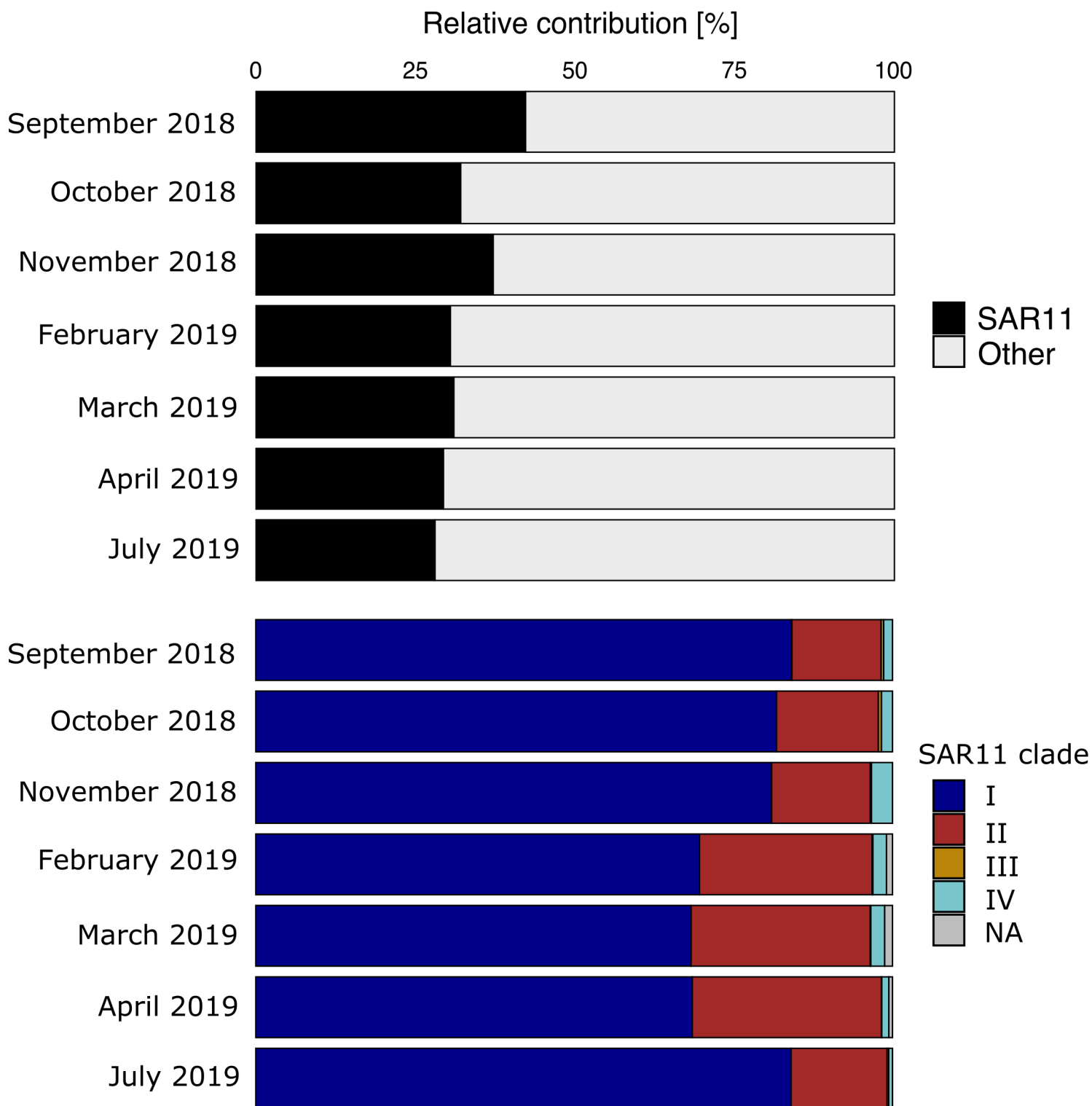

Supplement: Supplementary file 10 — Supplementary Figure 9 [file 41396_2020_872_MOESM10_ESM.pdf]

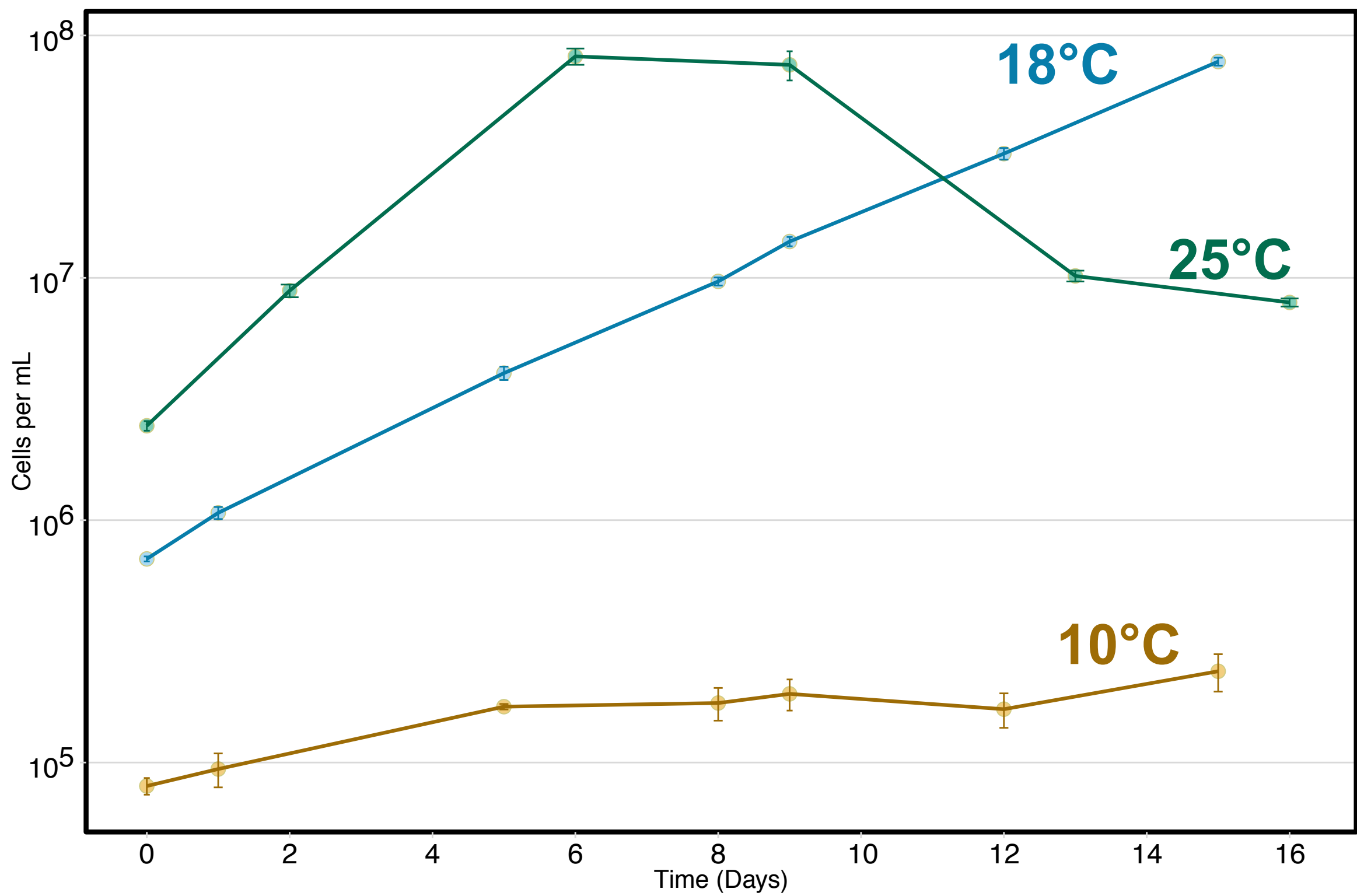

Supplement: Supplementary file 11 — Supplementary Figure 10 [file 41396_2020_872_MOESM11_ESM.pdf]

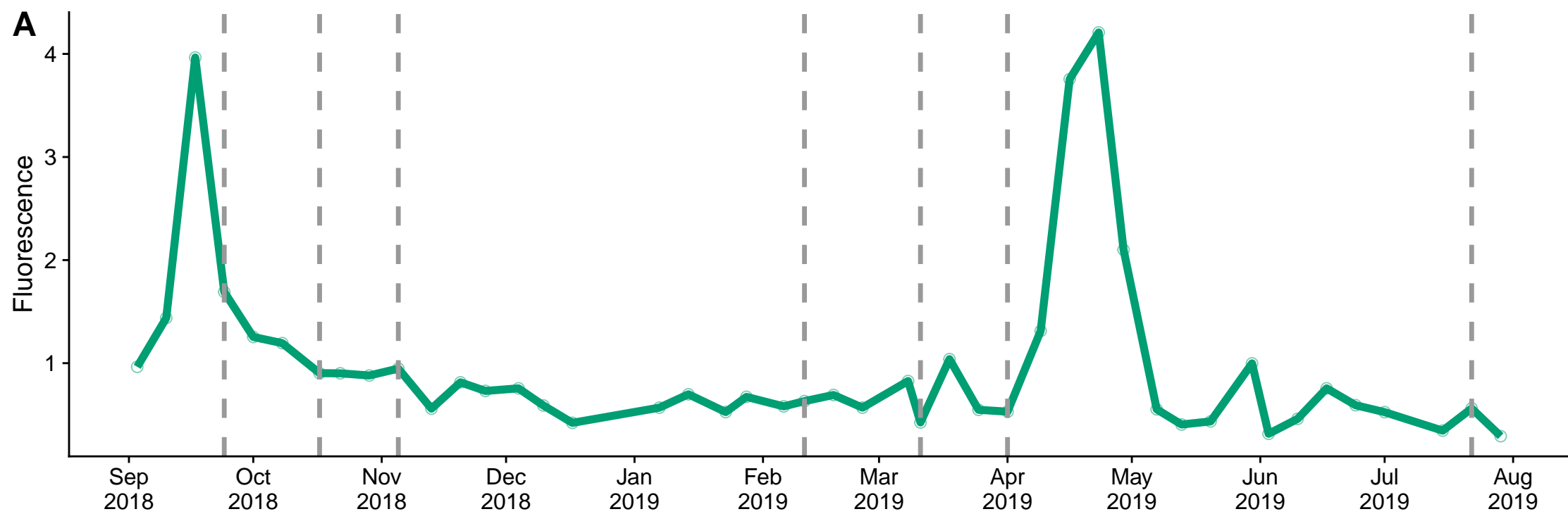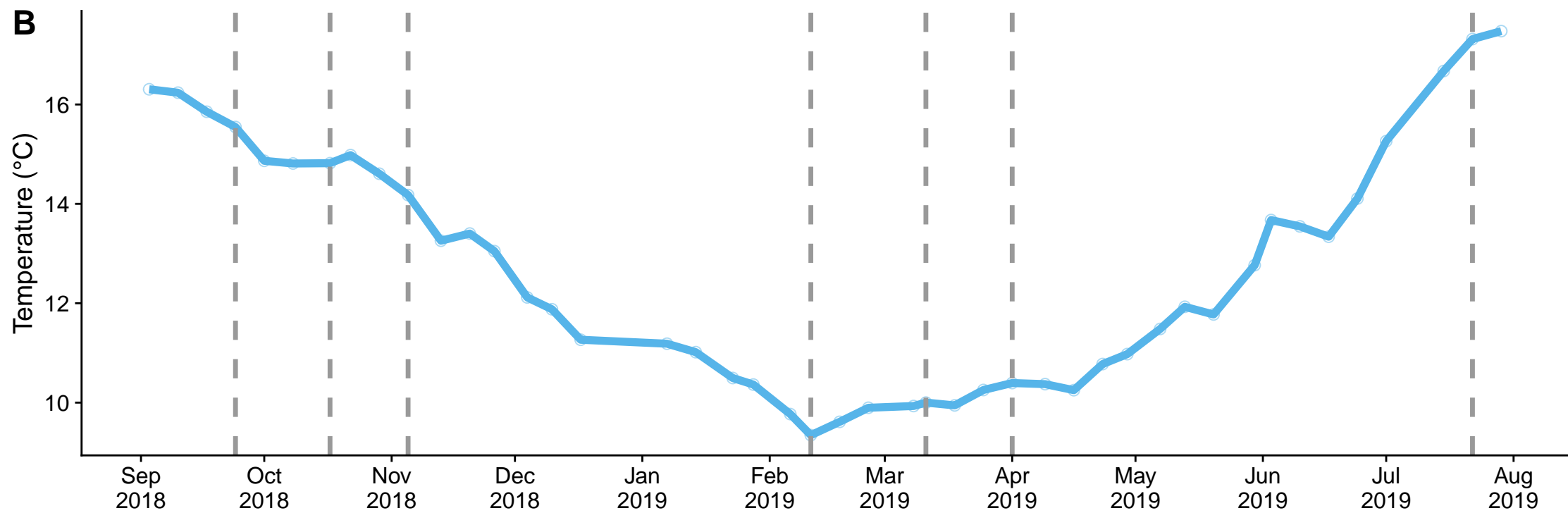

Supplement: Supplementary file 12 — Supplementary Figure 11 [file 41396_2020_872_MOESM12_ESM.pdf]

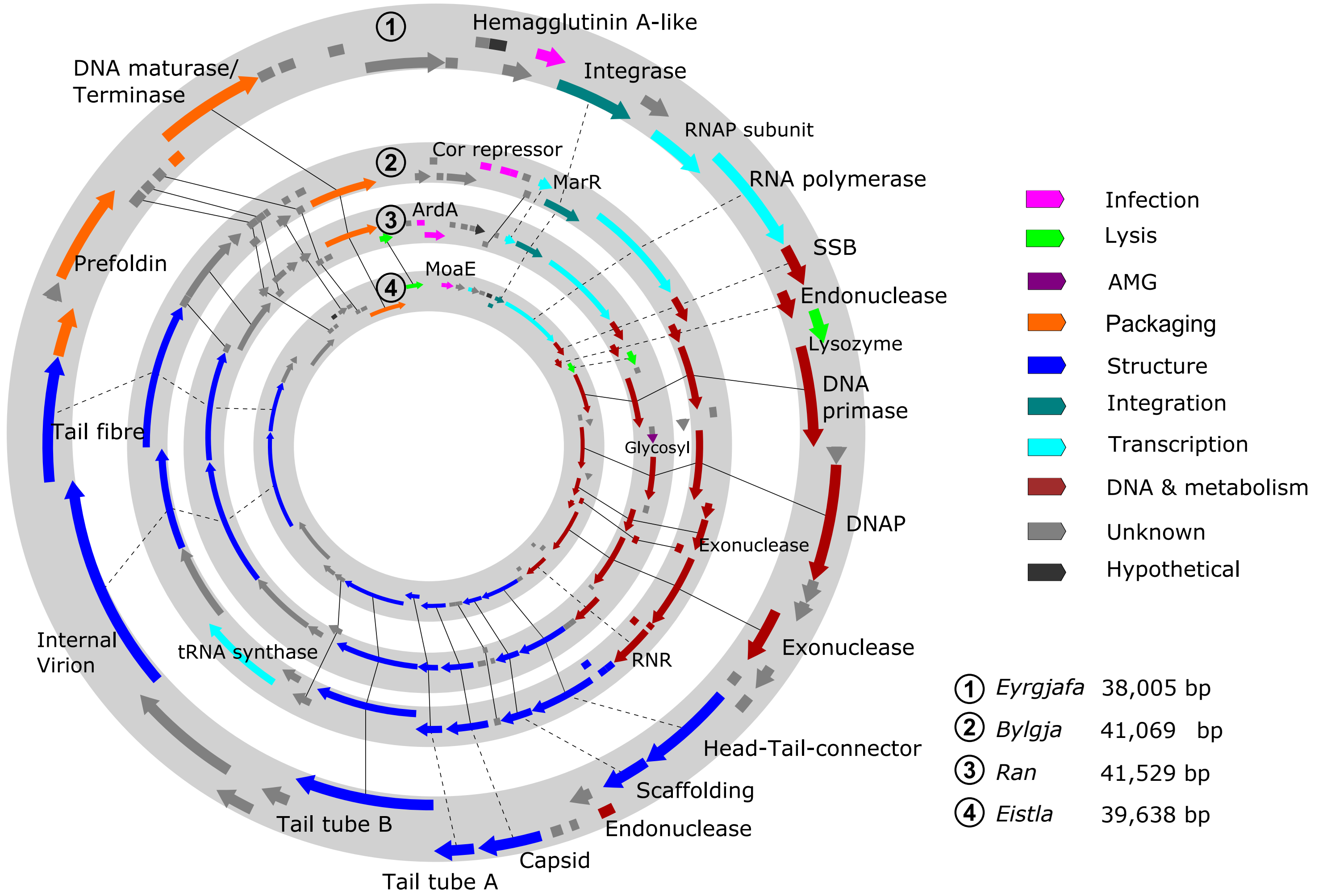

Supplement: Supplementary file 13 — Supplementary Figure 12 [file 41396_2020_872_MOESM13_ESM.pdf]

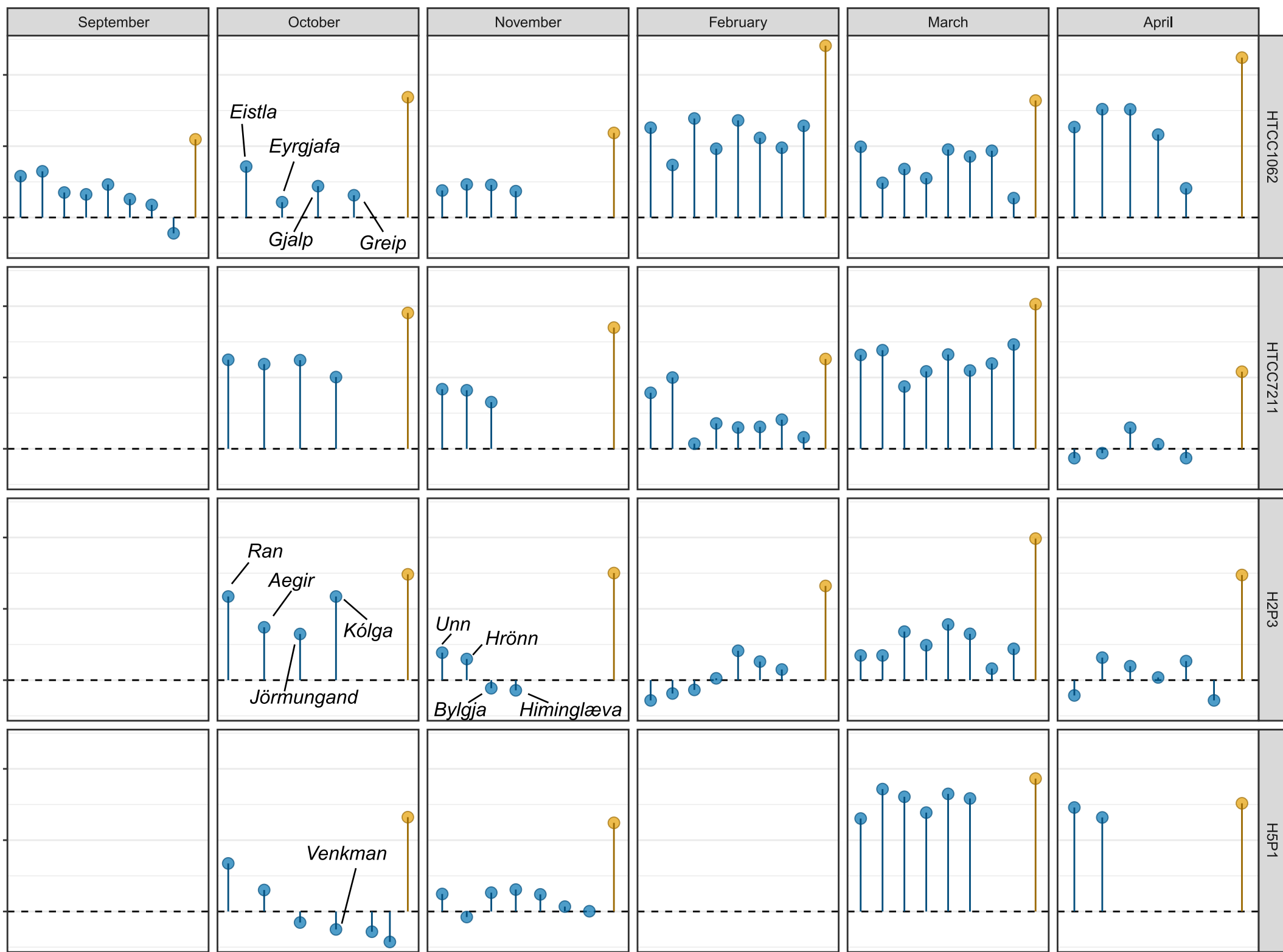

Supplement: Supplementary file 14 — Supplementary Figure 13 [file 41396_2020_872_MOESM14_ESM.pdf]
