# Supplementary material for: Efficient dilution-to-extinction isolation of novel virus–host model systems for fastidious heterotrophic bacteria
Source: ISME J. 2021 Jan 25;15(6):1585–98. doi: 10.1038/s41396-020-00872-z (PMC8163748; doi:10.1038/s41396-020-00872-z)
Supplement: Supplementary file 15 — Supplementary Table 1 [file 41396_2020_872_MOESM15_ESM.docx]

|  | **C6P1** | **D12P1** | **H5P1** | **HTCC2181** | **KB13** | **HIMB624** | **MBRSH7** |
| --- | --- | --- | --- | --- | --- | --- | --- |
| **C6P1** |  | 98.24 | 98.31 | 96.17 | 95.00 | 94.10 | 95.06 |
| **D12P1** | 98.24 |  | 98.83 | 96.62 | 95.39 | 94.50 | 95.46 |
| **H5P1** | 98.31 | 98.83 |  | 97.79 | 96.55 | 95.80 | 96.62 |
| **HTCC2181** | 96.17 | 96.62 | 97.79 |  | 96.49 | 95.40 | 96.55 |
| **KB13** | 95.00 | 95.39 | 96.55 | 96.49 |  | 99.70 | 99.93 |
| **HIMB624** | 94.10 | 94.50 | 95.80 | 95.40 | 99.70 |  | 99.60 |
| **MBRSH7** | 95.06 | 95.46 | 96.62 | 96.55 | 99.93 | 99.60 |  |
