# Supplementary material for: Efficient dilution-to-extinction isolation of novel virus–host model systems for fastidious heterotrophic bacteria
Source: ISME J. 2021 Jan 25;15(6):1585–98. doi: 10.1038/s41396-020-00872-z (PMC8163748; doi:10.1038/s41396-020-00872-z)
Supplement: Supplementary file 16 — Supplementary Table 2 [file 41396_2020_872_MOESM16_ESM.docx]

| **Collection Time** | **Collection Date** | **Temperature (°C)** | **Fluorescence** | **Depth (m)** | **Density** | **Salinity (psu)** | **CStarTr0** | **Par** | **Oxy** |
| --- | --- | --- | --- | --- | --- | --- | --- | --- | --- |
| 10:21:14 | Sept 24 2018 | 15.546 | 1.6909 | 5 | 1025.8704 | 34.9962 | 85.2961 | 0.4093 | 239.93 |
| 09:20:37 | Oct 17 2018 | 14.8181 | 0.9033 | 5 | 1025.7093 | 34.5798 | 69.7736 | 2.1833 | 236.3 |
| 10:27:16 | Nov 05 2018 | 14.1766 | 0.9475 | 5 | 1026.2688 | 35.126 | 88.7715 | 0.6285 | 240.45 |
| 10:17:04 | Feb 11 2019 | 9.3449 | 0.6315 | 5 | 1026.4935 | 34.2436 | 79.7166 | 1.6396 | 269 |
| 10:13:55 | Mar 11 2019 | 10.001 | 0.5425 | 5 | 1026.98864 | 35.0152 | 89.5834 | 0.6723 | 267.41 |
| 09:21:13 | Apr 01 2019 | 10.394 | 0.5298 | 5 | 1026.9579 | 35.0672 | 89.4219 | 0.4996 | 270.53 |
| 09:20:27 | Jul 22 2019 | 17.3155 | 0.5612 | 5 | 1025.6336 | 35.2245 | 94.7492 | 0.1291 | 238 |
| 09:09:43 | Aug 12 2019 | 16.9301 | 0.5437 | 5 | 1025.7526 | 35.2596 | 92.0995 | 0.2526 | 242.96 |
