# Supplementary material for: Efficient dilution-to-extinction isolation of novel virus–host model systems for fastidious heterotrophic bacteria
Source: ISME J. 2021 Jan 25;15(6):1585–98. doi: 10.1038/s41396-020-00872-z (PMC8163748; doi:10.1038/s41396-020-00872-z)
Supplement: Supplementary file 17 — Supplementary Table 3 [file 41396_2020_872_MOESM17_ESM.docx]

| **Water Sample** | **Target host** | **Rounds of Enrichment** |
| --- | --- | --- |
| **September** | *Pelagibacter ubique* HTCC1062 | 2 |
| **October** | *Pelagibacter bermudensis* HTCC7211 | 3 |
| **October** | *Pelagibacter ubique* HTCC1062 | 3 |
| **October** | P*elagibacter sp*. H2P3α | 3 |
| **October** | *Methylophilales sp.* H5P1 | 3 |
| **November** | *Pelagibacter bermudensis* HTCC7211 | 2 |
| **November** | *Pelagibacter ubique* HTCC1062 | 2 |
| **November** | P*elagibacter sp*. H2P3α | 2 |
| **November** | *Methylophilales sp.* H5P1 | 2 |
| **February** | *Pelagibacter bermudensis* HTCC7211 | 1 |
| **February** | *Pelagibacter ubique* HTCC1062 | 1 |
| **February** | P*elagibacter sp*. H2P3α | 1 |
| **February** | *Methylophilales sp.* H5P1 | 2 |
| **March** | *Pelagibacter bermudensis* HTCC7211 | 1 |
| **March** | *Pelagibacter ubique* HTCC1062 | 1 |
| **March** | P*elagibacter sp*. H2P3α | 1 |
| **March** | *Methylophilales sp.* H5P1 | 2 |
| **April** | *Pelagibacter bermudensis* HTCC7211 | 1 |
| **April** | *Pelagibacter ubique* HTCC1062 | 1 |
| **April** | P*elagibacter sp*. H2P3α | 1 |
| **April** | *Methylophilales sp.* H5P1 | 2 |
| **July** | *Methylophilales sp.* D12P1 | 2 |
